# A blast fungus zinc-finger fold effector binds to a hydrophobic pocket in host Exo70 proteins to modulate immune recognition in rice

**DOI:** 10.1073/pnas.2210559119

**Published:** 2022-10-17

**Authors:** Juan Carlos De la Concepcion, Koki Fujisaki, Adam R. Bentham, Neftaly Cruz Mireles, Victor Sanchez de Medina Hernandez, Motoki Shimizu, David M. Lawson, Sophien Kamoun, Ryohei Terauchi, Mark J. Banfield

**Affiliations:** ^a^Department of Biochemistry and Metabolism, John Innes Centre, Norwich, NR4 7UH, United Kingdom;; ^b^Division of Genomics and Breeding, Iwate Biotechnology Research Center, Iwate, 024-0003, Japan;; ^c^The Sainsbury Laboratory, University of East Anglia, Norwich, NR4 7UH, United Kingdom;; ^d^Laboratory of Crop Evolution, Graduate School of Agriculture, Kyoto University, Kyoto, 606-8501, Japan

**Keywords:** effector, plant immunity, NLR, Exocyst

## Abstract

Exocytosis plays an important role in plant–microbe interactions, in both pathogenesis and symbiosis. Exo70 proteins are integral components of the exocyst, an octameric complex that mediates tethering of vesicles to membranes in eukaryotes. Although plant Exo70s are known to be targeted by pathogen effectors, the underpinning molecular mechanisms and the impact of this interaction on infection are poorly understood. Here, we show the molecular basis of the association between the effector AVR-Pii of the blast fungus *Maganaporthe oryzae* and rice Exo70 alleles OsExo70F2 and OsExo70F3, which is sensed by the immune receptor pair Pii via an integrated RIN4/NOI domain. The crystal structure of AVR-Pii in complex with OsExo70F2 reveals that the effector binds to a conserved hydrophobic pocket in Exo70, defining an effector/target binding interface. Structure-guided and random mutagenesis validates the importance of AVR-Pii residues at the Exo70 binding interface to sustain protein association and disease resistance in rice when challenged with fungal strains expressing effector mutants. Furthermore, the structure of AVR-Pii defines a zinc-finger effector fold (ZiF) distinct from the MAX (Magnaporthe Avrs and ToxB-like) fold previously described for a majority of characterized *M*. *oryzae* effectors. Our data suggest that blast fungus ZiF effectors bind a conserved Exo70 interface to manipulate plant exocytosis and that these effectors are also baited by plant immune receptors, pointing to new opportunities for engineering disease resistance.

## Significance

Plant diseases destroy ∼20 to 30% of annual crop production, contributing to global food insecurity. Discovering how pathogen effectors target host proteins to promote virulence is essential for understanding pathogenesis and can be used for developing disease-resistant crops. Here, we reveal the structural basis of how an effector from the blast pathogen (AVR-Pii) binds a specific host target (rice Exo70) and how this underpins immune recognition. This has implications for understanding the molecular mechanisms of blast disease and for engineering new recognition specificities in plant immune receptors to confer resistance to a major crop pathogen.

Exocytosis is a cellular pathway in which membrane-bound vesicles are delivered from intracellular compartments to the plasma membrane for the release of their contents into the extracellular space (1). This pathway is essential for cell growth and division, as well as many other specialized processes that involve polarized secretion (1).

During exocytosis, a protein complex called the exocyst mediates the tethering of vesicles to the plasma membrane (2). The exocyst is an octamer formed by the proteins Sec3, Sec5, Sec6, Sec8, Sec10, Sec15, Exo70, and Exo84 (3), which assemble in a holocomplex (4–7) to control the spatiotemporal regulation of exocytosis, orchestrate cargo delivery, and mediate vesicle secretion (8–11). The exocyst complex is conserved in all eukaryotes. In yeast and mammals, the exocyst component Exo70 is encoded by a single gene (12). However, plant Exo70s have expanded dramatically, resulting in high diversity in protein sequence and multiple gene copies (12, 13). This suggests that Exo70 proteins may have functionally diversified and adopted specialized functions in plants (10). Indeed, different Exo70s are involved in diverse plant processes, including root development (14, 15), cell wall deposition (16, 17), symbiosis with arbuscular mycorrhiza (18), and cell trafficking pathways distinct from exocytosis, such as autophagy (19–21).

Specific plant Exo70 proteins have been associated with disease resistance to pathogens and pests (13, 22–25). Like other components of cellular pathways involved in homeostasis and/or signaling, the exocyst complex is targeted by plant pathogens to promote disease and, in some cases, is actively monitored by the immune system. For example, Exo70 proteins can be guarded by plant receptors of the NLR (nucleotide-binding, leucine-rich repeat) superfamily (26–28) and have been shown to interact with RIN4, a well-known regulator of plant immunity (29, 30) that is also targeted by effectors from diverse pathogens (31, 32). Both Exo70 and RIN4 domains are also found as integrated domains in plant NLRs (13, 33–36), suggesting the importance of these two proteins in disease and plant defense.

The blast fungus pathogen *Magnaporthe oryzae* delivers effectors into the host to alter cellular processes, aiding successful colonization (37, 38). Genome sequencing has uncovered hundreds of putative effectors harbored by this pathogen (39). However, only a small subset of these proteins have been functionally characterized to date. One such effector, AVR-Pii, interacts with two rice Exo70 subunits, OsExo70F2 and OsExo70F3, suggesting that the pathogen may target exocyst-mediated trafficking as a virulence-associated mechanism (28).

AVR-Pii was cloned alongside blast effectors AVR-Pik and AVR-Pia by association genetics (40). AVR1-CO39, AVR-Pik, and AVR-Pia are founding members of the MAX (Magnaporthe Avrs and ToxB-like) effector family (41), and studies on these effectors have been instrumental in defining the role of unconventional integrated domains in plant NLRs (42–49). Additional studies focused on these effectors and their cognate immune receptors have enabled engineering of bespoke immune responses to pathogen effectors (50–54). Despite the knowledge provided by structure/function studies of AVR-Pik and AVR-Pia, AVR-Pii has remained somewhat understudied.

Comprising only 70 residues, AVR-Pii is substantially smaller than AVR-Pik or AVR-Pia (40) and was not predicted to be a member of the MAX effector family (41). AVR-Pii has been reported to associate with only two alleles of the 47 members of the rice Exo70 family (12), OsExo70F2 and OsExo70F3 (28). These alleles share 72% sequence identity, while it is more common for Exo70 alleles to share only 30% sequence identity. This specificity suggests that AVR-Pii may target particular processes carried out by exocyst complexes harboring these Exo70 alleles. Despite the low overall sequence identity, Exo70 proteins from phylogenetically distinct organisms share a common fold (55–57). Therefore, AVR-Pii may exploit subtle structural differences to achieve this high interaction specificity. However, the molecular details of such stringent effector specificity are unknown. AVR-Pii is recognized by a rice disease-resistance gene pair named Pii, which is composed of the genetically linked genes Pii-2 and Pii-1 (58). This recognition requires at least OsExo70F3 (28), and the association of AVR-Pii with OsExo70F3 is monitored by Pii through an unconventional RIN4/NOI domain integrated in the sensor NLR Pii-2 (59). However, the precise mechanism of recognition remains obscure.

We focused on elucidating the molecular basis of *M*. *oryzae* AVR-Pii interaction with rice Exo70 proteins. By determining the crystal structure of the effector in complex with OsExo70F2, we defined a previously uncharacterised effector/target binding interface. We revealed that AVR-Pii adopts a zinc-finger fold (ZiF) that has not been reported previously for plant pathogen effectors (60) and is distinct from the MAX fold found in the *M*. *oryzae* effectors whose structure is known to date (41). We then used structure-informed and random mutagenesis to dissect how the Exo70/AVR-Pii interface underpins effector binding, exploring the basis of effector specificity. Finally, we correlated Exo70/AVR-Pii binding with Pii-mediated resistance in rice, further strengthening the link between host virulence–associated targets and immune regulation. The exocyst complex is a target of diverse plant pathogen effectors and is linked to resistance against pathogens and pests, suggesting a role as a hub in pathogenesis and plant defense. Our study expands our understanding of the molecular mechanisms used by plant pathogen effectors to target host proteins and may enable new approaches to the engineering of disease resistance.

## Results

### AVR-Pii Binds OsExo70F2 and OsExo70F3 with High Affinity and Specificity.

**Fig. 1.**
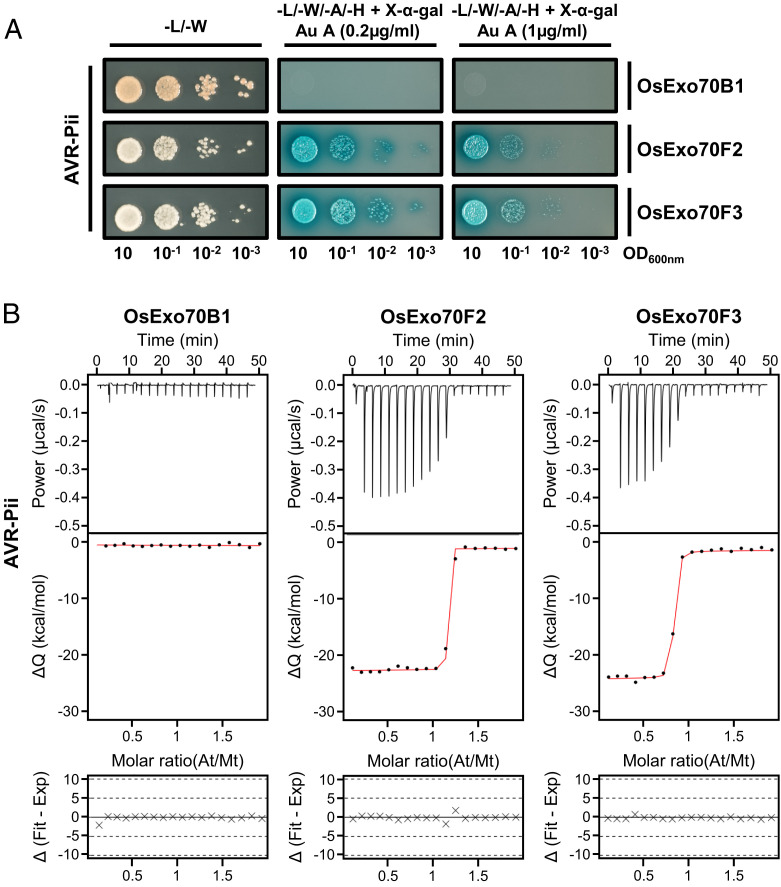
AVR-Pii binds specifically to rice Exo70F2 and Exo70F3 in yeast and in vitro. (*A*) Y2H assay of AVR-Pii with rice OsExo70B1, OsExo70F2, and OsExo70F3. *Left*, control plate for yeast growth. *Right*, quadruple-dropout media supplemented with X-α-gal and increasing concentrations of aureobasidine A (Au A). Growth and development of blue coloration in the selection plate are both indicative of protein-protein interactions. OsExo70 proteins were fused to the GAL4 DNA binding domain and AVR-Pii to the GAL4 activator domain. Each experiment was repeated a minimum of three times, with similar results. (*B*) Binding of AVR-Pii to OsExo70 proteins determined by ITC. *Upper*, heat differences upon injection of AVR-Pii into the cell containing the respective OsExo70 allele. *Middle*, integrated heats of injection (dots) and best fit (solid line) to a single-site binding model calculated using AFFINImeter ITC analysis software (78). *Bottom*, difference between the fit to a single-site binding model and the experimental data; closer to zero indicates stronger agreement between the data and the fit. The experiments shown are representative of three replicates. Other replicates for each experiment are shown in *SI Appendix*, Fig. S5. The thermodynamic parameters obtained in each experiment are presented in *SI Appendix*, Table S1.

To explore a detailed understanding of the interaction between AVR-Pii and rice Exo70s, we performed a Yeast-2-Hybrid assay (Y2H) coexpressing the effector with the rice Exo70 alleles OsExo70B1, OsExo70F2, and OsExo70F3. Consistent with previous work (28), we observed yeast growth and the development of blue coloration with X-α-gal, both readouts of protein-protein interactions, for AVR-Pii coexpressed with OsExo70F2 or OsExo70F3, but not with OsExo70B1 (Fig. 1*A*). This confirms that AVR-Pii specifically interacts with these Exo70 alleles. Growth of yeast was also clearly observed at elevated concentrations of aureobasidin A (Fig. 1*A*), suggesting that the association of AVR-Pii with OsExo70F2 and OsExo70F3 is robust.

To test for effector/target interactions in vitro, we optimized a pipeline to produce and purify rice Exo70 subunits OsExo70B1, OsExo70F2, and OsExo70F3 by heterologous expression in *Escherichia coli* (*SI Appendix*, Fig. S1; details in *SI Appendix*, *SI Materials and Methods*). Analytical gel filtration analysis of purified rice Exo70 alleles showed that proteins with truncated N-terminal domains elute as monodisperse peaks, suggesting they are suitable for further biophysical experiments (*SI Appendix*, Fig. S2). Likewise, we purified the effector domain of AVR-Pii (residues 20 to 70), adapting a protocol previously used for the purification of the blast effector AVR-Pik (*SI Appendix*, Fig. S3; details in *SI Appendix*, *SI Materials and Methods*). The molecular mass of the effector was confirmed by intact mass spectrometry (*SI Appendix*, Fig. S4).

To investigate the strength of binding between AVR-Pii and Exo70 alleles, we used isothermal titration calorimetry (ITC). We measured heat differences (indicative of protein-protein interactions) after titration of the AVR-Pii effector into a solution containing purified rice Exo70 proteins and used this information to calculate K*_d_* values for the interaction. These experiments showed AVR-Pii binds to both OsExo70F2 and OsExo70F3 with nanomolar affinity (Fig. 1*B* and *SI Appendix*, Fig. S5 and Table S1). No interaction was detected between AVR-Pii and OsExo70B1 (Fig. 1*B* and *SI Appendix*, Fig. S5 and Table S1), confirming the high specificity of the binding observed in Y2H.

In summary, we confirmed that AVR-Pii is a selective effector that binds to a specific subset of allelic rice Exo70s with high affinity.

### Crystal Structure of AVR-Pii in Complex with OsExo70F2.

After confirming that AVR-Pii binds to OsExo70F2 and OsExo70F3 in vitro, we showed that an OsExo70/AVR-Pii complex can be reconstituted and purified to homogeneity (*SI Appendix*, Fig. S6). A reconstituted OsExo70F2/AVR-Pii complex was stable and could reach high concentrations. Using this sample, we obtained protein crystals that diffracted X-rays to 2.7 Å resolution at the Diamond Light Source (Oxford, United Kingdom). Details of the X-ray data collection, structure solution, and structure completion are given in *SI Appendix*, *SI Materials and Methods*, and Table S2.

**Fig. 2.**
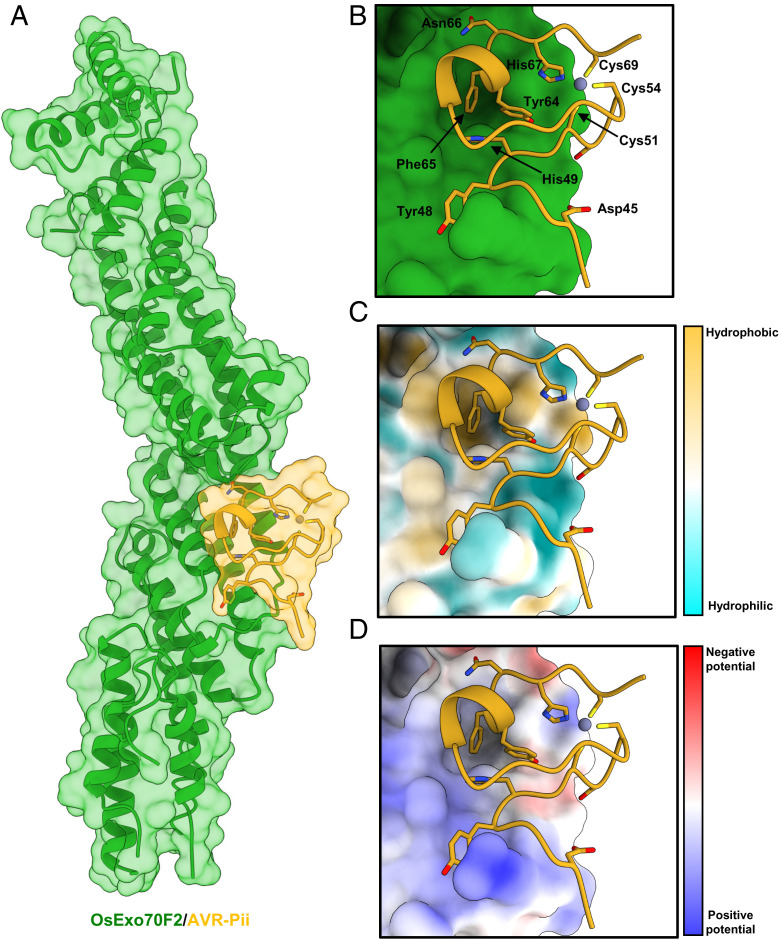
The crystal structure of OsExo70F2 in complex with AVR-Pii reveals hydrophobic residues dominate the interaction interface. (*A*) Schematic representation of OsExo70F2 in complex with AVR-Pii. Both molecules are represented as cartoon ribbons, with the molecular surface also shown and colored as labeled in green and yellow for OsExo70F2 and AVR-Pii, respectively. (*B*) Close-up view of the interaction interface between OsExo70F2 and AVR-Pii. OsExo70F2 is presented as a solid surface, with the effector as cartoon ribbons and side chains displayed as a cylinder for AVR-Pii–interacting residues (Asp45, Tyr48, His49, Tyr64, Phe65, and Asn66) in addition to the residues coordinating the Zn^2+^ atom (Cys51, Cys54, His67, and Cys69). (*C*) OsExo70F2 surface hydrophobicity representation at the AVR-Pii interaction interface; residues are colored depending on their hydrophobicity from light blue (low) to yellow (high). (*D*) Representation of OsExo70F2 surface electrostatic potential at the AVR-Pii interaction interface; residues are colored depending on their electrostatic potential from dark blue (positive) to red (negative). AVR-Pii residues 20 to 43 were not observed in the electron density used to derive the structure.

In the crystal structure, AVR-Pii and OsExo70F2 form a 1:1 complex (Fig. 2*A*). OsExo70F2 adopts an elongated rod-like shape formed by the stacking of four domains (domains A to D), with 16 α-helices in total (annotated α1 to α16) arranged in four-helix bundles (*SI Appendix*, Fig. S7*A*). Despite significant differences in sequence, the OsExo70F2 structure closely resembles the fold of the *Arabidopsis* AtExo70A1 protein (Protein Data Bank [PDB] ID 4L5R) (57) and the mouse MmExo70 protein (PDB ID 2PFT) (56), which can be aligned to the OsExo70F2 structure with an rmsd of 1.13 and 1.00 Å across 188 and 177 pruned atom pairs, respectively, as calculated with ChimeraX (61) (*SI Appendix*, Fig. S7*B*).

Consistent with existing Exo70 structures, the N terminus of OsExo70F2 was disordered, and the first residue modeled in the electron density was Ser85. Furthermore, several additional loop regions were disordered, including those connecting the helices α2 and α3 (residues 130 to 156), α3 and α4 (231 to 256), α6 and α7 (330 and 331), α10 and α11 (461 to 482), α13 and α14 (572 to 598), and α15 and α16 (648 to 659).

Following placement of OsExo70F2, we identified an electron density consistent with the sequence of AVR-Pii and were able to build residues 44 to 70 of the effector (residues 20 to 43 were not observed in the electron density). This C-terminal region of AVR-Pii revealed a fold for an *M*. *oryzae* effector based on a zinc-finger motif (Fig. 2 and *SI Appendix*, Fig. S8). This fold is sustained by AVR-Pii residues Cys51, Cys54, His67, and Cys69, which coordinate a Zn^2+^ atom (Fig. 2*B* and *SI Appendix*, Fig. S8). A structural similarity search performed with PDBeFold (62) revealed the AVR-Pii structure is most similar to LIM domain zinc fingers with a motif of C-X_2_-C-X_12_-H-X-C; however, it lacks a second zinc binding motif commonly found in this class of domains. We refer to the AVR-Pii fold as ZiF and note that this three-dimensional structure has not been previously reported to our knowledge for other plant pathogen effectors (60) and is distinct from the MAX fold found for other *M*. *oryzae* effectors whose structures are known (41).

### AVR-Pii Interacts with OsExo70F2 via a Hydrophobic Pocket.

The binding interface between OsExo70F2 and AVR-Pii is well resolved in the electron density. AVR-Pii locates to an amphipathic surface formed at the junction of OsExo70F2 domains B and C (Fig. 2 and *SI Appendix*, Fig. S8), with residues from helices α8, α9, and α10 contributing to the effector binding interface (Fig. 2*B* and *SI Appendix*, Fig. S8). Analysis of the complex using QtPISA (63) reveals both hydrophobic and hydrogen bond interactions in the complex, with a remarkable 20 of the 27 AVR-Pii residues (74%) observed for the effector in the crystal structure involved in contacts with OsExo70F2 (*SI Appendix*, Figs. S9 and S10). Furthermore, molecular lipophilicity potential and Coulombic electrostatic potential (calculated with ChimeraX (61)) reveal distinct hydrophobic and charged regions on the surface of OsExo70F2 surrounding the AVR-Pii binding interface (Fig. 2 *C* and *D*). The most striking feature of the OsExo70F2/AVR-Pii interface is a hydrophobic pocket formed by OsExo70F2 residues Phe416, Leu420, Met437, Tyr440, and Val441, which accommodates AVR-Pii Phe65 and, to a lesser extent, Tyr64 (Fig. 2 *B*–*D*).

### Amino Acid Variation in the OsExo70 Hydrophobic Pocket Underpins AVR-Pii Binding Specificity.

To better understand how AVR-Pii achieves a high binding specificity toward different Exo70 alleles, we analyzed conservation of residues at the OsExo70/AVR-Pii binding interface using Consurf (64). Unexpectedly, this analysis showed significant conservation in the residues at the AVR-Pii interface, with the residues surrounding the hydrophobic pocket showing limited variability (*SI Appendix*, Fig. S11). We then modeled rice OsExo70B1 using AlphaFold2 (65), as implemented in ColabFold (66) (*SI Appendix*, Fig. S12), to observe whether structural homology could help us understand specificity. While the N-terminal region of OsExo70B1 could not be accurately resolved, AlphaFold2 produced a high confidence model for the domains present in the OsExo70F2/AVR-Pii complex, including the effector binding interface. Side-by-side comparison of the sequence and structure of OsExo70F2 with the model of OsExo70B1 showed small differences in the residues forming the hydrophobic pocket, with OsExo70F2 Phe416, Val419, Leu420, and Met437 replaced by Leu405, Leu408, Ile-409, and Ile426 at equivalent positions in OsExo70B1 (*SI Appendix*, Fig. S13*A*). While these polymorphisms do not appear to alter the overall hydrophobicity or electrostatic potential at the effector binding interface (*SI Appendix*, Fig. S13), the hydrophobic pocket of OsExo70F2 is not observed in the OsExo70B1 model (*SI Appendix*, Figs. S13 and S14). Modeling of Exo70F2 and Exo70F3 using AlphaFold2 showed that this software can predict the hydrophobic pocket identified in our crystal structure, suggesting that the lack of a pocket in the Exo70B1 model is likely correct (*SI Appendix*, Fig. S14). This suggests that AVR-Pii residues Tyr64 and Phe65 could not be accommodated, resulting in the lack of binding to OsExo70B1 (*SI Appendix*, Figs. S13 and S14). Sequence alignment of rice Exo70 proteins showed that all alleles present differences at the equivalent residues forming the hydrophobic pocket in OsExo70F2 (*SI Appendix*, Fig. S15). Therefore, we conclude that AVR-Pii specificity is underpinned by small changes in the Exo70 binding interface that dramatically alter binding affinity.

### Mutations at the Exo70/AVR-Pii Interface Prevent Binding.

Prior to obtaining the structure of the OsExo70F2/AVR-Pii complex, we used random mutagenesis coupled with Y2H to identify AVR-Pii residues involved in binding to OsExo70 proteins (*SI Appendix*, Fig. S16). Using this approach, we obtained five independent AVR-Pii mutants within the effector domain, named M1 to M5 (*SI Appendix*, Fig. S16*A*). These mutants showed reduced (M2) or abrogated (M4 and M5) binding to OsExo70F3 (*SI Appendix*, Fig. S16*B*). Western blot analysis showed that M2 and M5 displayed a lower protein accumulation in yeast (*SI Appendix*, Fig. S16*C*). Because M2 and M5 carry mutations in Zn^2+^ binding residues (Cys54Arg in M2 and Cys51Arg in M5), it is likely these affect protein folding and protein stability. AVR-Pii M4 was the only mutant displaying lack of binding without compromised effector accumulation. This mutant harbors two residue changes, Arg43Ser and Tyr64Asp. We therefore generated single mutants Arg43Ser, Arg43Ala, Tyr64Asp, and Tyr64Ala to investigate the contribution of these residues to the binding to OsExo70F3. Y2H assays show that only Tyr64Asp prevented AVR-Pii binding to OsExo70F3 (*SI Appendix*, Fig. S16*D*), and none of these mutations affected protein accumulation (*SI Appendix*, Fig. S16*E*).

**Fig. 3.**
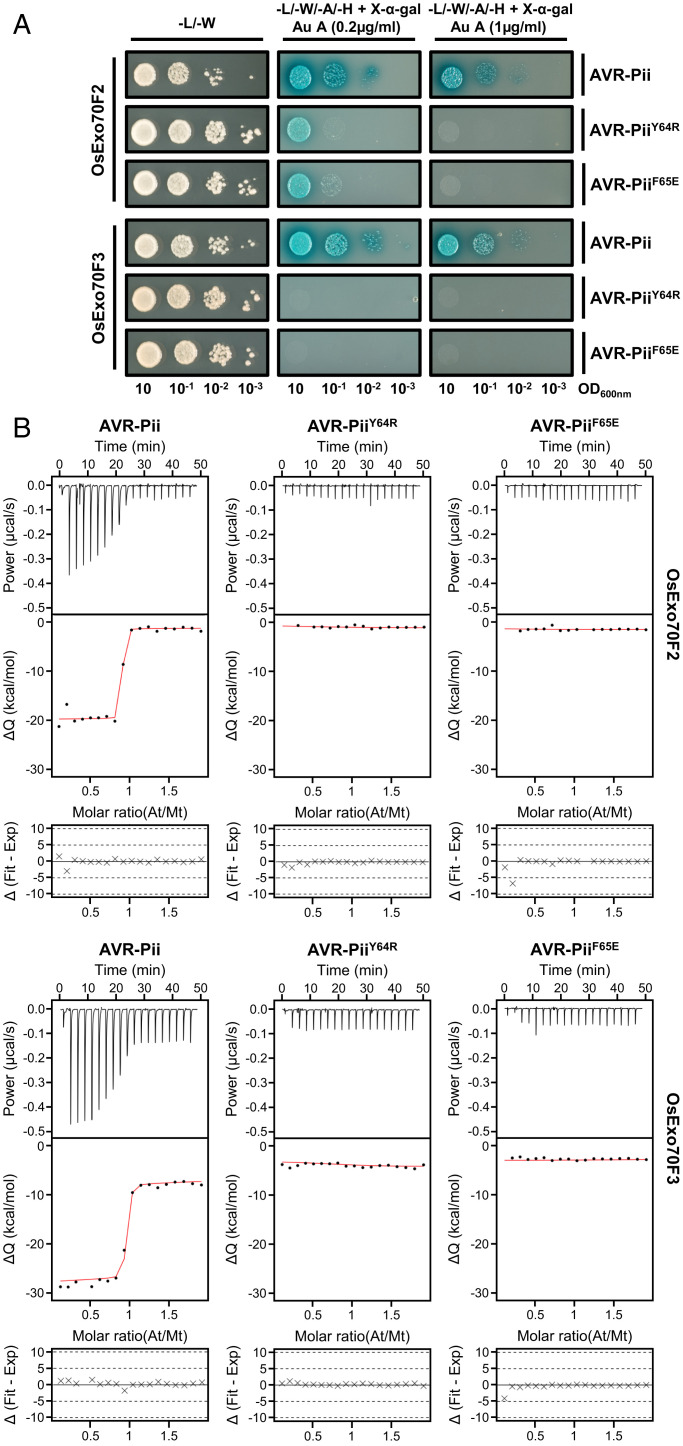
Mutations at the AVR-Pii binding interface perturb interactions with rice Exo70 proteins. (*A*) Y2H assay of AVR-Pii mutants Tyr64Arg and Phe65Glu with rice OsExo70F2 and OsExo70F3. *Left*, control plate for yeast growth. *Right*, quadruple-dropout media supplemented with X-α-gal and increasing concentrations of aureobasidine A (Au A). Growth and development of blue coloration in the selection plate are both indicative of protein-protein interactions. Wild-type AVR-Pii is included as positive control. Exo70 proteins were fused to the GAL4 DNA binding domain and AVR-Pii to the GAL4 activator domain. Each experiment was repeated a minimum of three times, with similar results. (*B*) Binding of AVR-Pii mutants Tyr64Arg and Phe65Glu to rice OsExo70F2 and OsExo70F3 determined by ITC. Wild-type AVR-Pii was included as positive control. *Upper*, heat differences upon injection of AVR-Pii mutants into the cell containing the respective OsExo70 allele. *Middle*, integrated heats of injection (dots) and best fit (solid line) to a single-site binding model calculated using AFFINImeter ITC analysis software (78). *Bottom*, difference between the fit to a single-site binding model and the experimental data; closer to zero indicates stronger agreement between the data and the fit. Panels are representative of three replicates. Other replicates for each experiment are shown in *SI Appendix*, Figs. S18 and S19. The thermodynamic parameters obtained in each experiment are presented in *SI Appendix*, Table S3.

Then, based on the crystal structure, we designed point mutants in AVR-Pii residues Tyr64 (Tyr64Arg) and Phe65 (Phe65Glu) because these were the dominant residues revealed at the interface. Y2H assays showed that AVR-Pii Tyr64Arg and Phe65Glu mutations severely affected binding to OsExo70F2 and prevented binding to OsExo70F3 (Fig. 3*A*). These mutations did not affect protein accumulation of the effector in yeast cells (*SI Appendix*, Fig. S17). To extend the Y2H analysis, we expressed and purified AVR-Pii Tyr64Arg and Phe65Glu mutants and tested their ability to bind OsExo70F2 and OsExo70F3 by ITC. Consistent with the Y2H assays, Tyr64Arg and Phe65Glu mutations affected binding of AVR-Pii to OsExo70F2 and OsExo70F3, with essentially no binding observed using this technique (Fig. 3*B* and *SI Appendix*, Figs. S18 and S19 and Table S3). Together, these experiments confirmed the AVR-Pii residues that locate to the OsExo70F2 hydrophobic pocket are essential for target binding and effector specificity.

### Mutations at the Exo70/AVR-Pii Interface Abrogate Pii-Mediated Resistance to Rice Blast.

Having identified residues that prevent Exo70/AVR-Pii binding by Y2H and in vitro, we transformed the *M*. *oryzae* Sasa2 strain (which lacks AVR-Pii) with wild-type AVR-Pii and the Tyr64Arg and Phe65Glu mutants to observe the impact on resistance mediated by the Pii NLR pair. For this assay, different independent Sasa2 transformants were recovered and their virulence tested in the susceptible rice cultivar Moukoto. We discarded noninfective transformants and performed RT-PCR to test for expression of the effector in the remaining strains (*SI Appendix*, Fig. S20).

**Fig. 4.**
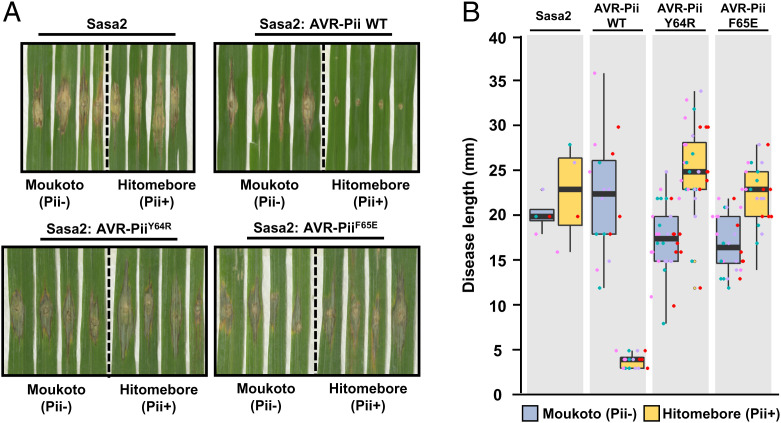
Mutations at the binding interface of AVR-Pii with OsExo70 abrogate recognition by Pii resistance in rice. (*A*) Rice leaf blade spot inoculation of transgenic *M*. *oryzae* Sasa2 isolates expressing AVR-Pii, AVR-Pii Tyr64Arg, or AVR-Pii Phe65Glu in rice cultivars Moukoto (Pii−) and Hitomebore (Pii+). For each experiment, representative images from replicates with independent *M*. *oryzae* transformants are shown. Wild-type (WT) *M*. *oryzae* isolate Sasa2 is included as control. Images for each replicate of AVR-Pii, AVR-Pii Tyr64Arg, and AVR-Pii Phe65Glu are presented in *SI Appendix*, Figs. S21–S23. (*B*) Measurement of vertical length of the disease lesion caused by *M*. *oryzae* Sasa2 as well as transgenic *M*. *oryzae* Sasa2 isolates harboring AVR-Pii, AVR-Pii Tyr64Arg, or AVR-Pii Phe65Glu in rice cultivars Moukoto (Pii−) and Hitomebore (Pii+). Lesions in rice cultivars Moukoto (Pii−) and Hitomebore (Pii+) are represented by blue and yellow boxes, respectively. For each isolate, a total of four biological replicates were performed, and the data are presented as box plots. The center line represents the median, the box limits are the upper and lower quartiles, and the whiskers extend to the largest value within Q1 − 1.5× the interquartile range (IQR) and the smallest value within Q3 + 1.5× IQR. All the data points are represented as dots with distinct colors for each biological replicate.

Infective strains expressing AVR-Pii wild type, Tyr64Arg, or Phe65Glu were then spot inoculated on rice cultivars Moukoto (lacking Pii resistance) and Hitomebore (harboring Pii resistance). The length of lesions was measured 10 d postinfection to assay the extent of disease progression (Fig. 4). As expected, all strains were virulent on the susceptible rice cultivar Moukoto, but the strains expressing wild-type AVR-Pii did not form expanded lesions on Hitomebore. However, we observed that AVR-Pii mutants that abrogate binding to OsExo70F2 and OsExo70F3 in Y2H and in vitro assays are not recognized in Hitomebore, with large disease lesions forming equivalent in size to those observed for untransformed Sasa2 (Fig. 4 and *SI Appendix*, Figs. S21–S23). These data corroborate the direct link between OsExo70/AVR-Pii binding and recognition by the rice NLR pair Pii.

## Discussion

Exocytosis has recently emerged as a conserved eukaryotic trafficking pathway with roles in plant immunity and symbiosis (18, 21–24). The exocyst complex is targeted by effectors from diverse pathogens (28, 67), and Exo70 domains have been integrated into plant NLRs (33, 34), likely to act as effector baits for the detection of pathogens by the immune system. Therefore, understanding the molecular and structural bases of manipulation of plant exocytosis by pathogen effectors has the potential to uncover new mechanisms of pathogen virulence and, ultimately, may pave new ways for engineering the outcome of plant–pathogen interactions (50, 52, 54, 68). In this study, we uncovered the molecular details of how the blast pathogen effector AVR-Pii binds to rice exocyst subunit Exo70, and we dissected the structural determinants of this interaction.

Although hundreds of putative effectors are encoded in pathogen genomes, only limited examples of plant pathogen effector-host target interactions have been dissected in molecular detail to date (69). Intriguingly, some pathogen effectors have evolved to target specific members of large host protein families, potentially to avoid compromising host cell viability. AVR-Pii is an example of an effector with a striking target specificity, because it was reported to associate with only two of 47 members of the rice Exo70 protein family (28). By obtaining the structure of the OsExo70F2/AVR-Pii complex, we revealed the molecular basis of such a high specificity. Surprisingly, the effector binds to a moderately conserved region of the Exo70 domain, but one that contains subtle differences between alleles (*SI Appendix*, Figs. S11 and S15). Therefore, AVR-Pii appears to have evolved high specificity by exploiting small differences at the Exo70 binding interface, specifically within a hydrophobic pocket that allows for the docking of the effector.

Although a precise function of the exocyst complex in plant immunity remains to be described, some Exo70 alleles have been shown to associate with RIN4, a well-known target of multiple pathogen effectors that is guarded by the plant immune system (29, 30). An increasing number of effectors have been reported to alter the Exo70-RIN4/NOI immune node (30), and much like RIN4, some Exo70s activate the plant immune system upon perturbation of their function (26).

The structure of AVR-Pii reported here reveals a protein fold for fungal effectors, based on a zinc-finger domain, that differs from the MAX fold shared by all the *M*. *oryzae* effectors whose structure is known to date (41, 43, 70, 71). Zinc-finger domains are abundant in nature and can be regularly found as single domains in larger multidomain proteins that are implicated in a variety of processes, from DNA interaction to signaling hubs and protein-protein scaffolds that regulate cellular functions, such as autophagy or G protein–coupled receptor signaling (72, 73). While the AvrP effector from Flax rust, *Melampsora lini*, also has been reported to have a ZiF (74), these two proteins share no structural similarity.

AVR-Pii targets OsExo70F2 and OsExo70F3, suggesting the effector may interfere with a function related to a unique role of these Exo70 alleles. However, any specific function of OsExo70F2 or OsExo70F3, compared with other rice Exo70 alleles, remains to be determined. Structural modeling based on the Cryo-EM model of the yeast exocyst (4) suggests that AVR-Pii would sit on the outer side of the complex, where it would not be expected to disrupt the association of the subunits forming subcomplex I (Sec3, Sec5, Sec6, and Sec8) or subcomplex II (Sec10, Sec15, Exo70, and Exo84) (*SI Appendix*, Fig. S24*A*). However, AVR-Pii is found close to the interface between Sec5 and Exo70, which may interfere with the necessary assembly of both exocyst subcomplexes into the holocomplex for the late stages of exocytosis (11) (*SI Appendix*, Fig. S24*B*). This suggests a function by which AVR-Pii may target assembly of exocyst complexes equipped with Exo70 subunits OsExo70F2 and OsExo70F3, but additional experiments are required to investigate this.

Plant immune receptors have been the focus of engineering to improve disease resistance to some of the most destructive pathogens of crops (75), with limited success. Recent studies of *M*. *oryzae* MAX effectors, particularly AVR-Pik and AVR-Pia, which target HMA domain–containing proteins (69, 76, 77) and are bound by integrated HMA domains in NLR receptors (44, 46, 47), have demonstrated proof of concept for engineering NLRs to generate expanded recognition profiles (50–54). Like HMA domains, Exo70s are found as integrated domains in some plant NLRs (33, 34). In addition to defining an effector fold and determining the structural basis of an effector/target interface, our results will help uncover new approaches for NLR engineering, for example, by repurposing integrated Exo70 domains to perceive different effectors.

## Materials and Methods

### Gene Cloning.

Detailed information for gene cloning is provided in *SI Appendix*, *SI Materials and Methods*.

### Protein Expression and Purification.

A full protocol for the heterologous expression and purification of rice Exo70 proteins and *M*. *oryzae* AVR-Pii effector is provided in *SI Appendix*, *SI Materials and Methods*.

### Crystallization, Data Collection, and Structure Solution.

Details for X-ray data collection, structure solution, and structure completion are given in *SI Appendix*, *SI Materials and Methods*.

### Protein–Protein Interaction.

#### Yeast-Two-Hybrid.

To detect protein-protein interactions between rice Exo70 proteins and AVR-Pii effectors in a Y2H system, we used the Matchmaker Gold System (Takara Bio USA) following a protocol adapted from De la Concepcion et al. (46), detailed in *SI Appendix*, *SI Materials and Methods*.

#### Isothermal Titration Calorimetry.

ITC experiments were performed using a MicroCal PEAQ-ITC (Malvern, United Kingdom). In each case, 300 μL OsExo70 at 10 μM was placed in the calorimetric cell and titrated with 100 μM AVR-Pii wild type or mutant in the syringe. Each run included a single injection of 0.5 μL followed by 18 injections of 2 μL each at intervals of 120 s with a stirring speed of 750 rpm. Data were processed with AFFINImeter ITC analysis software (78). ITC runs for wild type and mutants were done in triplicate at 25 °C in 20 mM Hepes (pH 7.5), 150 mM NaCl, and 5% (vol/vol) glycerol buffer supplemented with 1 mM TCEP.

### Rice Blast Infection Assay.

Conidial suspension (2 to 5 × 10^5^ conidia/mL) was prepared from the transgenic *M*. *oryzae* and used for leaf blade spot inoculation using rice cultivar Hitomebore (Pii+) and Moukoto (Pii−) as described previously (79). Disease lesions were photographed 10 d after inoculation, and vertical length was measured.

### RT-PCR.

For RT-PCR analysis, total RNA was extracted from the disease lesion–containing Moukoto leaves, and complementary DNA was synthesized using oligo dT primer. RT-PCR was performed using a specific primer set for AVR-Pii and for *M*. *oryzae* actin as control.

## Supplementary Material

Supplementary File

## Data Availability

All study data are included in the article and/or supporting information. Protein structure of the OsExo70F2/AVR-Pii complex, and the data used to derive it, has been deposited at the Protein Data Bank (PDB) with accession code 7PP2 (80).
