# Supplementary material for: A blast fungus zinc-finger fold effector binds to a hydrophobic pocket in host Exo70 proteins to modulate immune recognition in rice
Source: Proc Natl Acad Sci U S A. 2022 Oct 17;119(43):e2210559119. doi: 10.1073/pnas.2210559119 (PMC9618136; doi:10.1073/pnas.2210559119)
Supplement: Supplementary File [file pnas.2210559119.sapp.pdf]

Supporting Information for
A blast fungus Zinc-finger fold effector binds to a hydrophobic pocket in host Exo70 proteins to modulate immune recognition in rice

Juan Carlos De la Concepcion, Koki Fujisaki, Adam R. Bentham, Neftaly Cruz Mireles, Victor Sanchez de Medina Hernandez, Motoki Shimizu, David M. Lawson, Sophien Kamoun, Ryohei Terauchi, Mark J. Banfield

Mark J. Banfield
Email: mark.banfield@jic.ac.uk

This PDF file includes:

Supporting text
Figures S1 to S24
Tables S1 to S3
SI References

Supplementary information

Extended materials and methods

Gene cloning

For protein production in *E. coli*, codon optimised AVR-Pii (amino acid residues Leu20 to Asn70) was synthesized (GenScript) and subsequently cloned into the pOPINM vector (1) using the In-Fusion cloning kit (Takara Bio USA). AVR-Pii Trp64Arg and Phe65Glu were synthesized as PCR products (Gblocks, IDT) and cloned into pOPINM in the same way. Truncated versions of rice Exo70 alleles OsExo70B1$^{\Delta 91}$, OsExo70F2$^{\Delta 83}$ and OsExo70F3$^{\Delta 93}$ were generated using standard molecular biology techniques from appropriate templates described by Fujisaki et al. (2) and cloned into pOPINS3C (1) using the In-Fusion cloning kit (Takara Bio USA).

For Y2H, wild-type and mutant AVR-Pii effectors (amino acid residues Leu20 to Asn70) were cloned in pGADT7 while full length CDS of rice Exo70 alleles OsExo70B1, OsExo70F2 and OsExo70F3 were cloned in pGBKT7. In both cases, plasmids were linearized by double digestion with EcoRI and BamHI (New England Biolabs) and genes of interest were introduced using the In-Fusion cloning kit (Takara Bio USA).

For random mutagenesis we used the Diversify PCR Random Mutagenesis Kit (Takara Bio USA) and subsequently cloned the mutagenized AVR-Pii PCR fragments in pGADT7 as described above.

To construct pCB1531 plasmids with AVR-Pii wild-type, Trp64Arg and Phe65Glu under control of pex22 promotor (3), coding sequences were amplified from appropriate templates by PCR using primer set KF1341f (5'GCTCTAGAAAAATGCAACTTTCCAAAATTAC3') and KF1295r (5'CGGGATCCTTAGTTGCATTTATGATT 3'). The PCR products were digested by BamHI and XbaI and inserted into the vector pCB1531-pex22p-EGFP (3) linearized with BamHI and XbaI. Resulting plasmids (pCB1531-pex22p-AVR-Pii-WT, Y64R and F65E) were transformed into *M. oryzae* Sasa2 strain lacking AVR-Pii gene as described previously (4).

Expression and purification of proteins for X-ray crystallography and in vitro binding studies

To enable the study of the OsExo70/AVR-Pii interactions in vitro, we produced stable rice OsExo70 proteins in *E. coli*. SUMO-tagged OsExo70 alleles with the predicted N-terminal α-helix truncated encoded in pOPINS3C were produced in E. coli Rosetta™ (DE3). Cell cultures were grown in autoinduction media (5) at 37°C for 5–7 hr and then at 16°C overnight. Cells were harvested by centrifugation and re-suspended in 50 mM Tris-HCl (pH 7.5), 500 mM NaCl, 50 mM glycine, 5% (vol/vol) glycerol, and 20 mM imidazole supplemented with EDTA-free protease inhibitor tablets (Roche). Cells were sonicated and, following centrifugation at 40,000xg for 30 min, the clarified lysate was applied to a HisTrap™ Ni2+-NTA column connected to an AKTA Xpress purification system (GE Healthcare). Proteins were step-eluted with elution buffer (50 mM Tris-HCl (pH7.5), 500 mM NaCl, 50 mM glycine, 5% (vol/vol) glycerol, and 500 mM imidazole) and directly injected onto a Superdex 200 26/60 gel filtration column pre-equilibrated with 20 mM HEPES (pH 7.5), 150 mM NaCl and 5% (vol/vol) glycerol supplemented with 1mM TCEP. Elution fractions were collected and evaluated by SDS-PAGE, revealing a band close to 70 kDa **(Figure S1a)**. Fractions were combined and incubated overnight with 3C protease (10 µg/mg fusion protein).

Rice Exo70 proteins were separated from the SUMO tag by passing the protein mixture solution through a HisTrap™ Ni2+-NTA column equilibrated with 50 mM Tris-HCl (pH 7.5), 500 mM NaCl, 50 mM glycine, 5% (vol/vol) glycerol, and 20 mM imidazole **(Figure S1b)**. Exo70 proteins were mainly present in the first and second wash-through **(Figure S1b)** from the column, whilst the SUMO tag was retained until the final elution with elution buffer **(Figure S1b)**. Fractions containing

Exo70 proteins were pooled together and concentrated for further purification and buffer exchange by gel filtration onto a Superdex 200 16/60 column pre-equilibrated with 20 mM HEPES (pH 7.5), 150 mM NaCl and 5% (vol/vol) glycerol supplemented with 1mM TCEP **(Figure S1c)**. Fractions containing purified Exo70 proteins were combined and concentrated for structural and biophysical studies.

For OsExo70 gel filtration analysis, a volume of 110 µl of each sample was separated at 4 °C on a Superdex 200 10/300 size exclusion column (GE Healthcare), pre-equilibrated in 20 mM HEPES (pH 7.5), 150 mM NaCl, 1 mM TCEP and 5% (vol/vol) glycerol and at a flow rate of 0.5 ml/min. Fractions of 0.5 ml were collected for analysis by SDS–PAGE.

MBP-tagged effector domain (amino acid residues 20 to 70) for wild-type AVR-Pii, Trp64Arg and Phe65Glu encoded by the pOPINM constructs were produced in *E. coli* SHuffle cells (6). Cell cultures were grown in autoinduction media (5) at 30°C for 5–7 hr and then at 16°C overnight. After harvest by centrifugation, cells were resuspended and disrupted as described above for OsExo70 expression.

The soluble fusion protein 6xHis:MBP:AVR-Pii was be purified from *E. coli* cell lysates by IMAC on a HisTrap™ Ni2+-NTA column connected to an AKTA Xpress purification system (GE Healthcare) coupled with gel filtration onto a Superdex 75 26/60 gel filtration column pre-equilibrated with 20 mM HEPES (pH 7.5), 150 mM NaCl and 5% (vol/vol) glycerol **(Figure S3a)**. The fractions containing the eluted protein were subsequently treated with 3C protease as before to remove the MBP tag. AVR-Pii was purified from the MBP solubility tag using HisTrap™ and MBPTrap™ (GE Healthcare) columns attached in tandem **(Figure S3b)**. Purified AVR-Pii was commonly present as a double band in the flow-through (FT) and wash-through (WT) from the columns **(Figure S3b)**.

The relevant fractions were concentrated and loaded onto a Superdex 75 16/60 gel filtration column for final purification and buffer exchange into 20 mM HEPES (pH 7.5), 150 mM NaCl and 5% (vol/vol) glycerol **(Figure S3c)**. Relevant fractions with purified AVR-Pii were concentrated as appropriate and used for structural and biophysical characterization.

The state of the protein was assessed by intact mass spectrometry, revealing a main peak with a molecular weight of 5677.68 Da, identical to that calculated for AVR-Pii **(Figure S4)**.

All protein concentrations were determined using a Direct Detect Infrared Spectrometer (Merck).

Crystallization, data collection and structure solution

For crystallization, OsExo70F2 (residues 84 to 689) in complex with AVR-Pii (residues 20 to 70) was concentrated to 6 mg/ml following gel filtration. Sitting drop vapor diffusion crystallization trials were set up in 96 well plates, using an Oryx nano robot (Douglas Instruments, United Kingdom), with drops comprised of 0.3 µl precipitant solution and 0.3 µl of the sample, and incubated at 20°C. After four to six days, protein crystals for the complex between OsExo70F2 and AVR-Pii appeared in the 0.3 M Ammonium iodide; 30% v/v PEG3350 condition of the PEG Suite screen (Qiagen). For data collection, all crystals were harvested using Litholoops (Molecular Dimensions) and flash-cooled in liquid nitrogen.

X-ray data were collected from a single crystal at the Diamond Light Source using beamline i03 (Oxford, UK) at 100 K and recorded on a Pilatus3 6M hybrid photon counting detector (Dectris). The data were processed using the xia2 pipeline (7) and CCP4 (8). To solve the structure of OsExo70F2/AVR-Pii complex, we used the Arabidopsis Exo70A1 (PDB ID: 4L5R) divided into three ensembles (Ensemble 1 residues 76 to 325; Ensemble 2 residues 326 to 474; Ensemble 3 residues 475 to 593) as a template for molecular replacement using PHASER (9). Once we obtained a solution, automated model building using BUCANNEER (10) was able to identify and build the AVR-Pii effector. The asymmetric unit of the crystal contains only a single copy of the OsExo70F2/AVR-Pii complex with a stoichiometry of 1:1. The final structure was obtained through

iterative cycles of model building and refinement using COOT (11), REFMAC5 (12), and ISOLDE (13) as implemented in the CCP4 suite (8) and ChimeraX (14). Structures were validated using the tools provided in COOT and MOLPROBITY (15).

Yeast-2-hybrid

The OsExo70 proteins encoded in pGBKT7 plasmids were co-transformed with AVR-Pii variants or mutants in pGADT7 into chemically competent Y2HGold cells (Takara Bio, USA) using a Frozen-EZ Yeast Transformation Kit (Zymo research).

Single colonies grown on selection plates were inoculated in 5 ml of SD -Leu -Trp overnight at 30 °C. Saturated culture was then used to make serial dilutions of OD_{600} 1, 0.1, 0.01, and 0.001. 5 µl of each dilution was spotted on a SD -Leu -Trp plate as a growth control, and on a SD -Leu -Trp -Ade -His plate containing X-α-gal and supplemented with 0.2 or 1 µg/ml Aureobasidin A (Takara Bio, USA). Plates were imaged after incubation for 60 – 72 h at 30 °C. Each experiment was repeated a minimum of three times, with similar results.

To confirm protein expression, total yeast extracts from transformed colonies were produced by boiling the cells for 10 min in LDS Runblue™ sample buffer (Expedeon). Samples were centrifugated and the supernatant was subjected to SDS-PAGE gels and western blot. The membranes were probed with anti-GAL4 DNA-BD (Sigma) for the OsExo70 proteins in pGBKT7 and with the anti-GAL4 activation domain (Sigma) antibodies for the AVR-Pii wild type and mutant effectors in pGADT7.

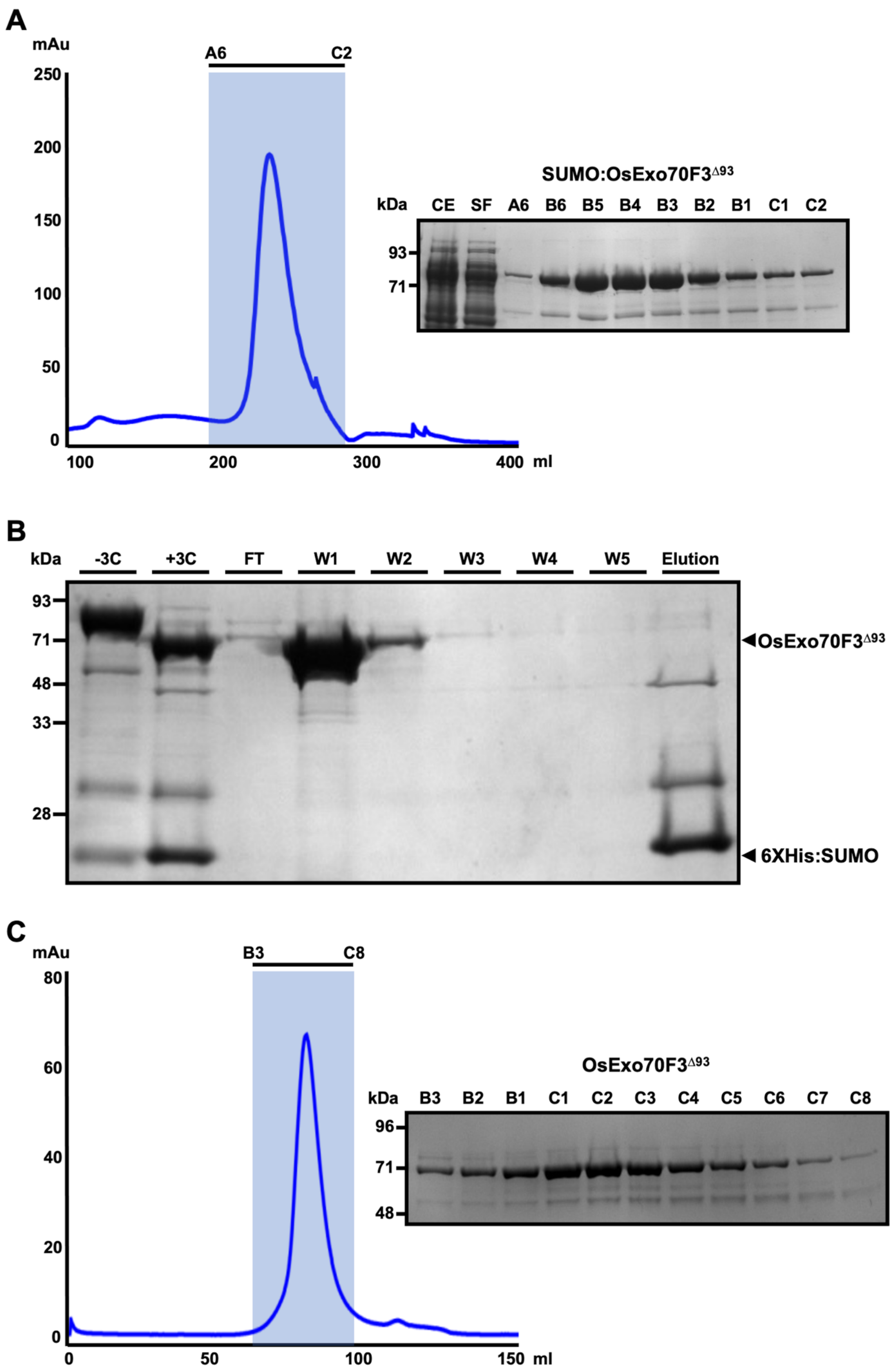

Fig. S1. Expression and purification of rice OsExo70 proteins. (A) Elution trace of SUMO:OsExo70F3$^{\Delta 93}$ after IMAC and gel filtration with selected fractions analyzed by SDS-PAGE. **(B)** SDS-PAGE analysis of fractions collected in the OsExo70F3$^{\Delta 93}$ HisTrap™ purification before and after cleaving the SUMO tag with 3C protease. OsExo70F3$^{\Delta 93}$ was successfully purified in the first and second wash fractions. The 6xHis:SUMO tag is released upon treatment with elution buffer. **(C)** Elution trace of OsExo70F3Δ93 after gel filtration and SDS-PAGE analysis of relevant fractions.

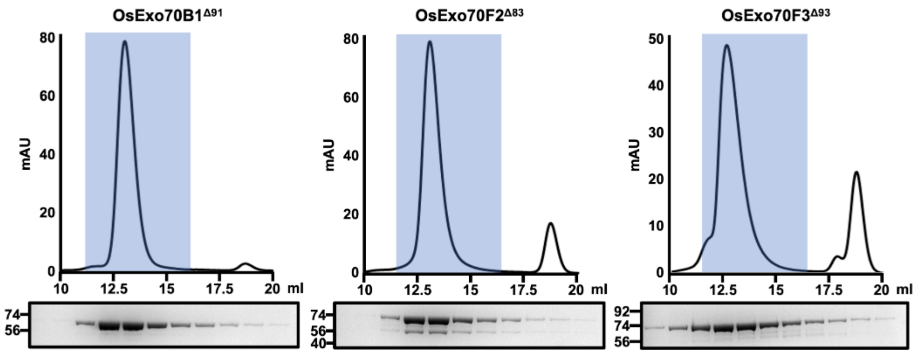

Fig. S2. Purified plant Exo70 proteins elute as single peaks in analytical gel filtration analysis. Elution traces of purified OsExo70B1$^{\Delta91}$, OsExo70F2$^{\Delta83}$ and OsExo70F3$^{\Delta93}$ in analytical gel filtration. SDS-PAGE analysis of the fractions highlighted in ice blue are shown below each trace.

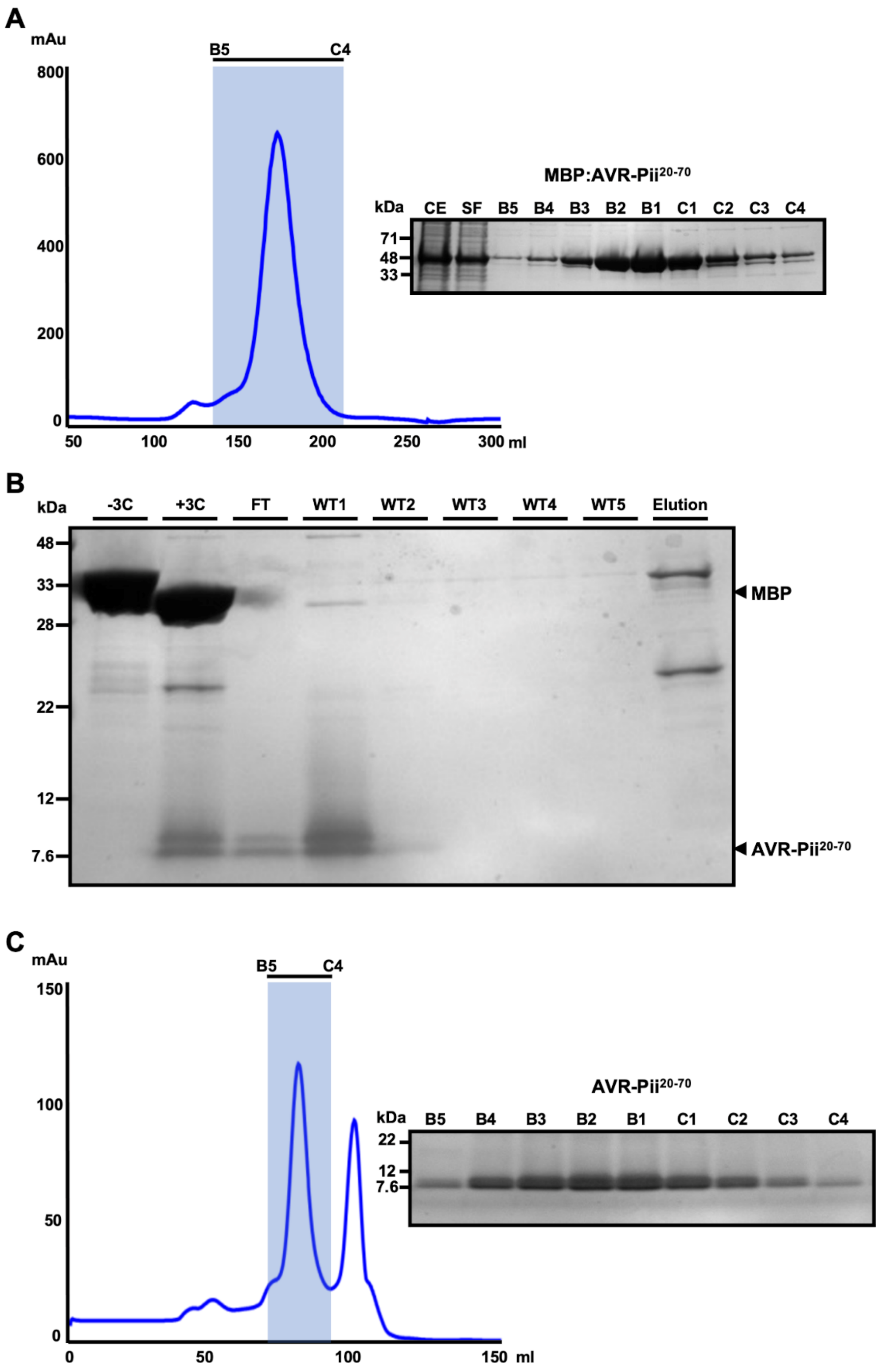

Fig. S3. Expression and purification of AVR-Pii. (A) Elution trace of MBP:AVR-Pii$^{20\text{-}70}$ after IMAC and gel filtration with selected fractions analyzed by SDS-PAGE. **(B)** SDS-PAGE analysis of fractions collected in the AVR-Pii$^{20\text{-}70}$ HisTrap™/ MBPTrap™ purification before and after cleaving the MBP tag with 3C protease. AVR-Pii$^{20\text{-}70}$ was successfully purified in the flow-through and first

wash fractions. **(C)** Elution trace of AVR-Pii$^{20-70}$ after gel filtration and SDS-PAGE analysis of relevant fractions.

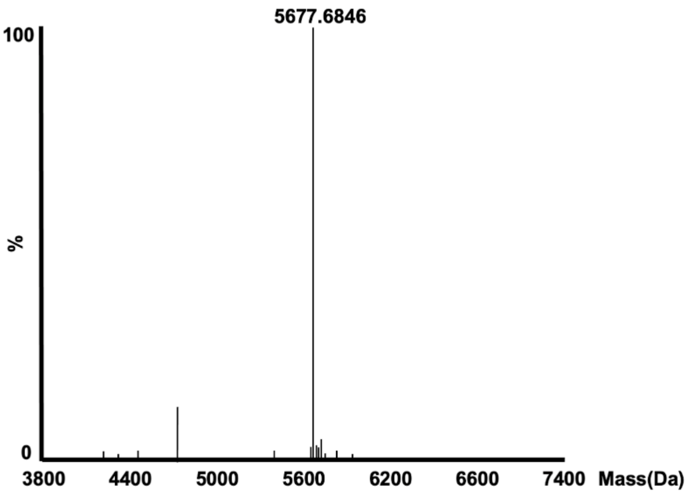

Fig. S4. Mass spectrometry analysis of purified AVR-Pii. Intact mass analysis of purified AVR-Pii$^{20\text{-}70}$ protein shows a main peak close to the predicted protein mass (5,675.0 Da).

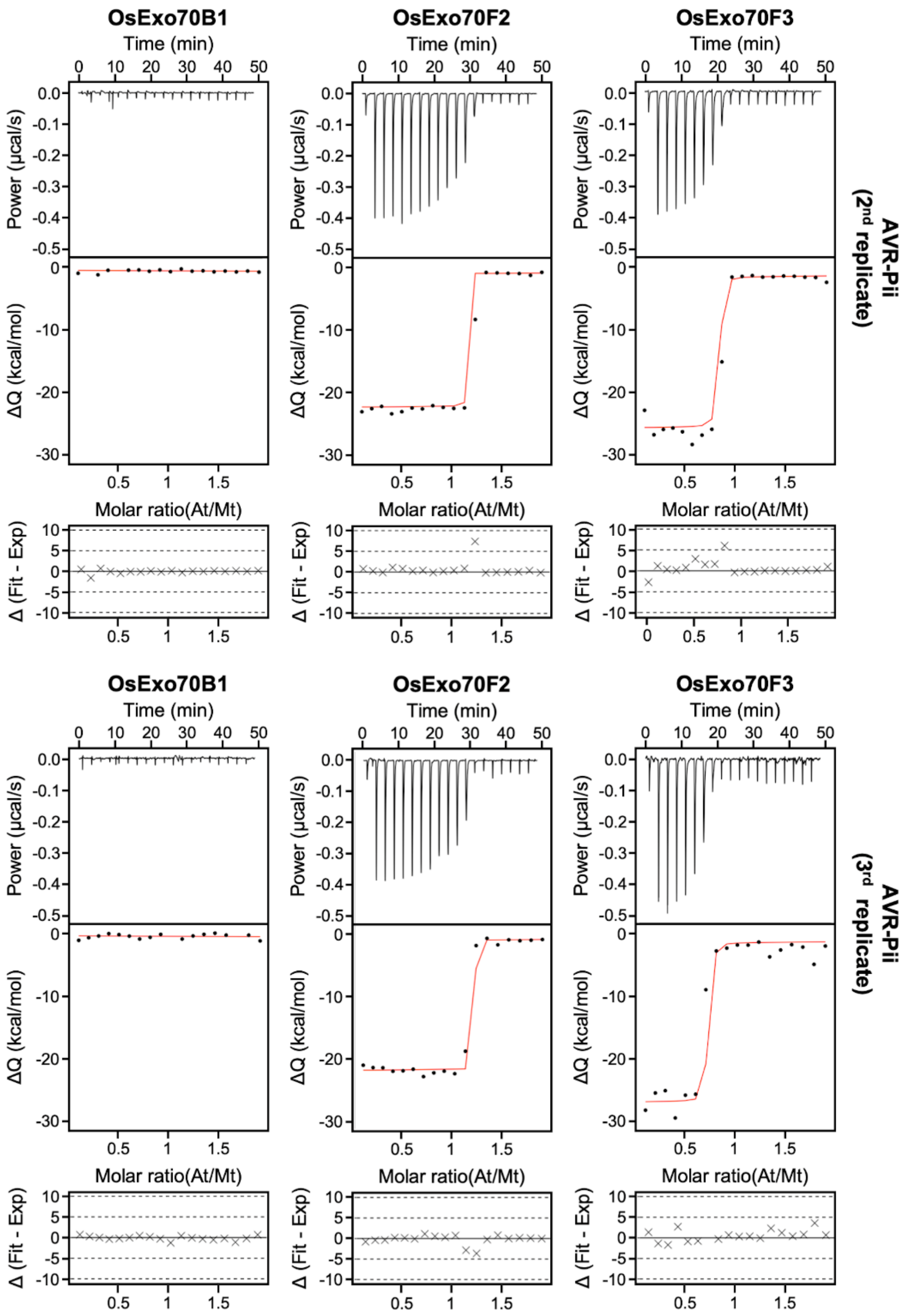

Fig. S5. Experimental replicates of AVR-Pii binding to OsExo70s measured by ITC. Binding of AVR-Pii to rice OsExo70 proteins determined by isothermal titration calorimetry (ITC). Upper panels show heat differences upon injection of AVR-Pii into the cell containing the respective OsExo70 allele. Middle panels show integrated heats of injection (dots) and the best fit (solid line) using to a single site binding model calculated using AFFINImeter ITC analysis software (16). Bottom panels represent the difference between the fit to a single site binding model and the experimental data; the closer to zero indicates stronger agreement between the data and the fit. The thermodynamic parameters obtained in each experiment are presented in **Table S1**.

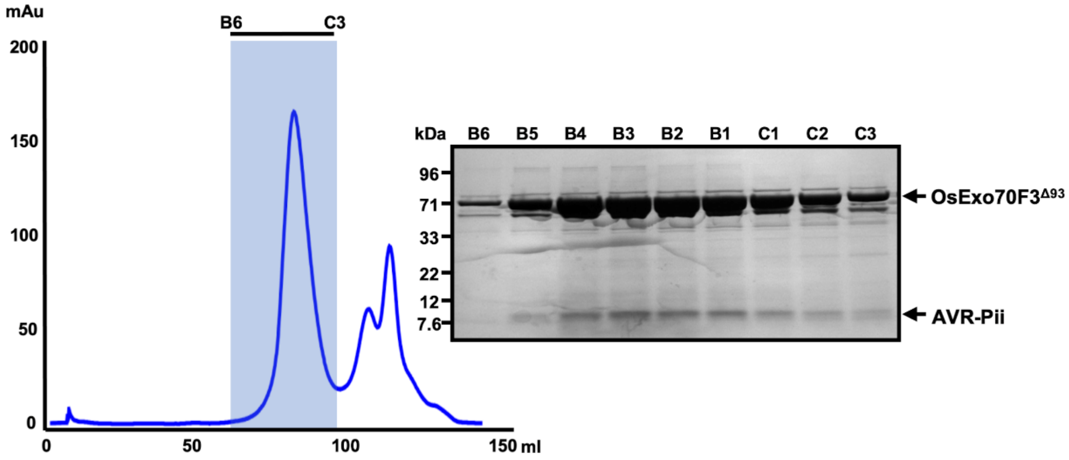

Fig. S6. The OsExo70/AVR-Pii complex can be reconstituted in vitro. Elution trace of reconstitution of OsExo70/AVR-Pii complex after gel filtration using OsExo70F3$^{\Delta 93}$ and AVR-Pii as example. Selected fractions were collected and analyzed by SDS-PAGE showing the presence of both proteins.

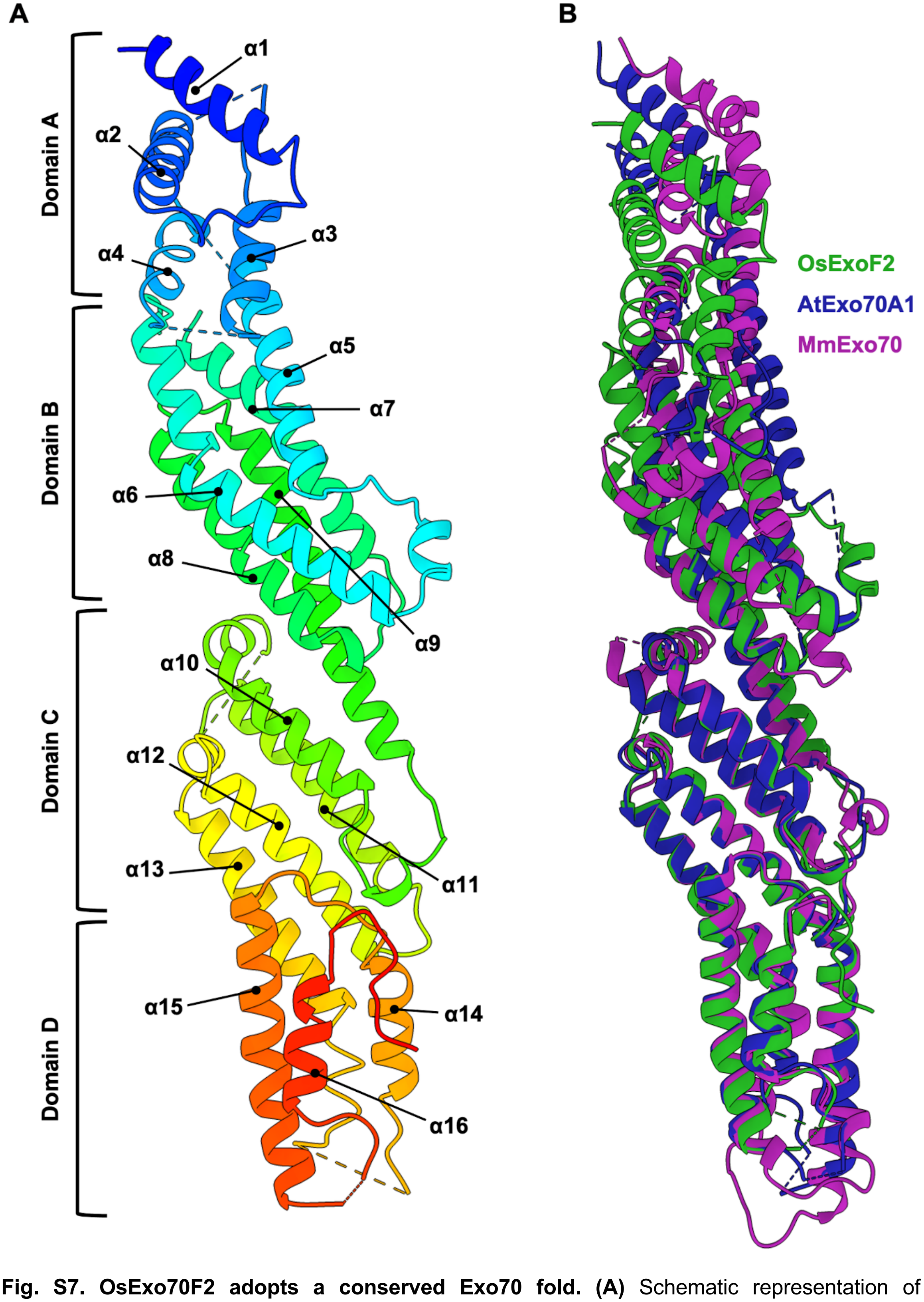

Fig. S7. OsExo70F2 adopts a conserved Exo70 fold. (A) Schematic representation of OsExo70F2 domains with α-helices as cartoon ribbons. **(B)** Superposition of the overall structures of rice OsExo70F2 (green), Arabidopsis AtExo70A1 (PDB ID: 4L5R) (17) (dark blue) and mouse

Exo70 (PDB ID: 2PFT) (18) (purple). Each Exo70 is colored as labelled and is shown in cartoon representation.

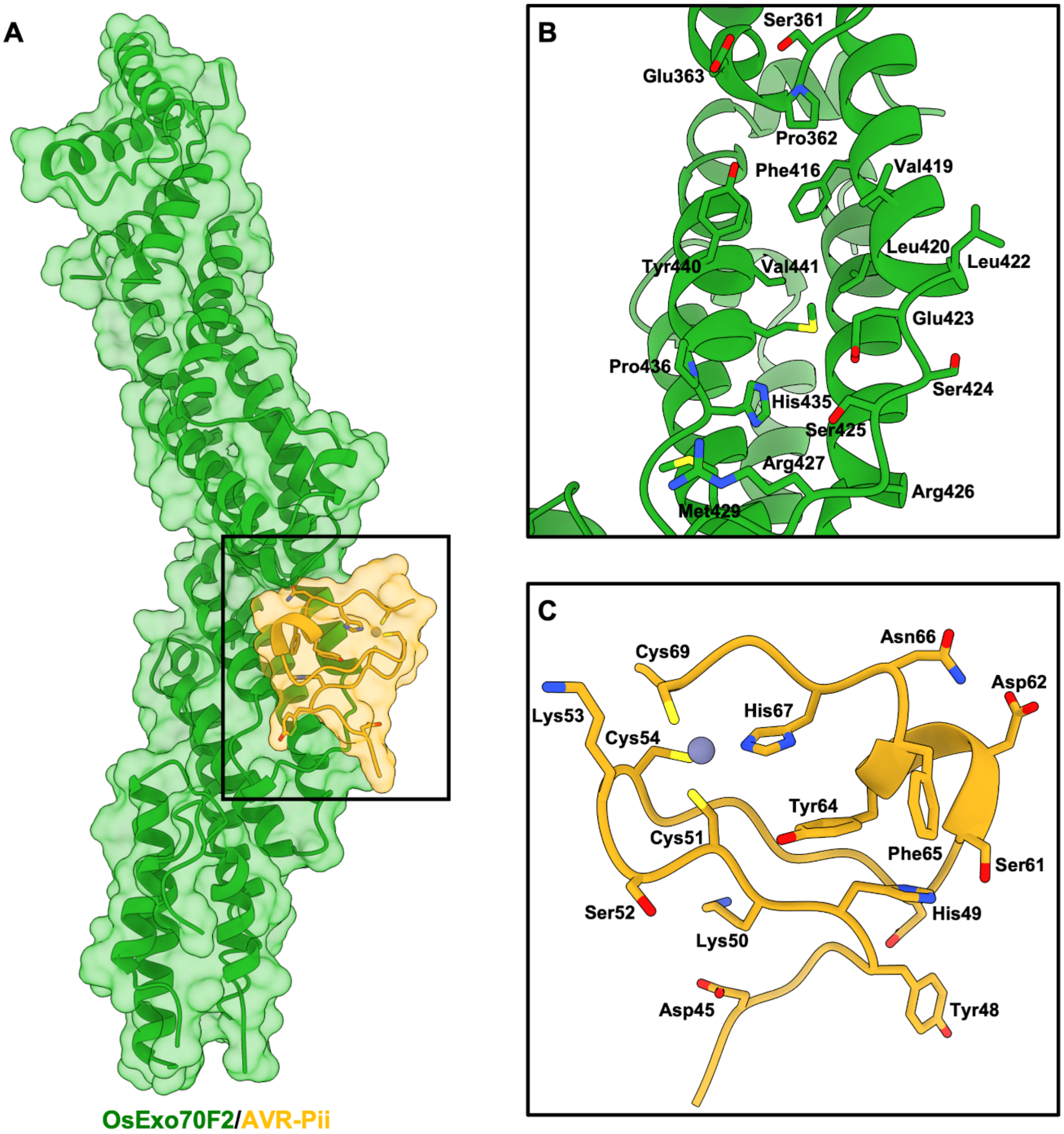

Fig. S8. Detailed view of residues at the OsExo70F2/AVR-Pii interface. (A) Schematic representation of OsExo70F2/AVR-Pii complex represented as cartoon ribbons with the molecular surface also shown and colored as labelled. The interaction interface is delimited by the black square. **(B)** Close-up view of residues comprising the OsExo70F2 interaction interface with AVR-Pii represented as cartoon ribbons. Residues forming the interaction interface are labelled with their side chains displayed as cylinders. **(C)** Close-up view of residues comprising the AVR-Pii interaction interface with OsExo70F2 represented as cartoon ribbons. Residues forming the interaction interface are labelled with their side chains displayed as cylinders. AVR-Pii residues 20 to 43 were not observed in the electron density used to derive the structure.

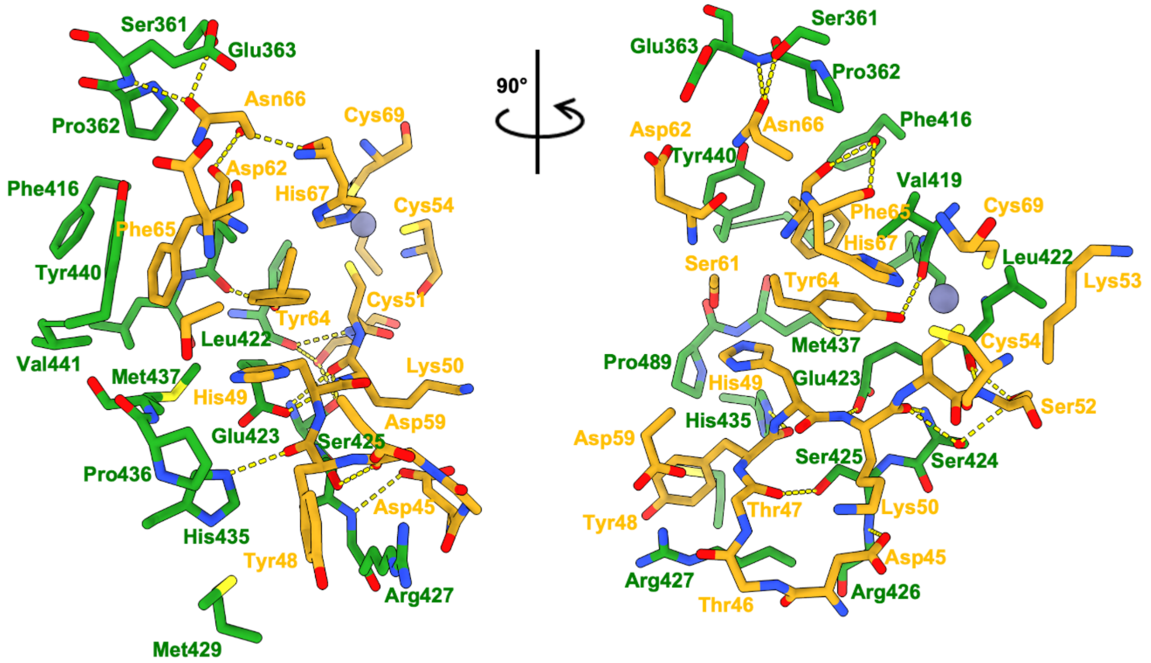

Fig. S9. Interfacing residues within the OsExo70F2/AVR-Pii complex. Residues at OsExo70F2/AVR-Pii interaction interface displayed as cylinders. Residues from OsExo70F2 and AVR-Pii are colored green or yellow, respectively. Hydrogen bonds/salt bridges are shown as yellow dashed lines.

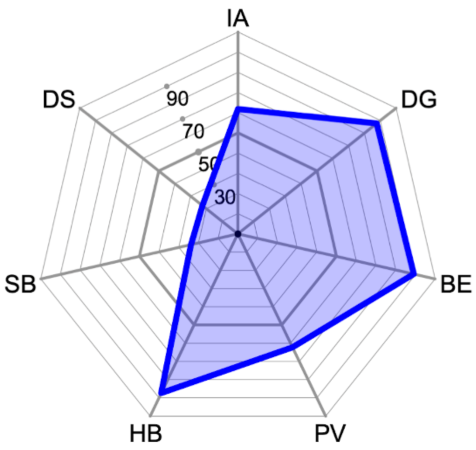

Interface Parameters

**Interface area (IA)**	834
**Delta G (DG)**	-11.7
**Binding energy (BE)**	-19.5
**Hydrophobic P-value (PV)**	0.1075
**Hydrogen bonds (HB)**	16
**Salt bridges (SB)**	2
**Disulfide bonds (DS)**	0

Interface Summary

	**AVR-Pii**	**Exo70-F2**
**Atoms in the interface**	90 (42.5%)	96 (2.7%)
**on the surface**	168 (79.2%)	2140 (60.7%)
**total**	212 (100.0%)	3526 (100.0%)
**Residues in the interface**	20 (74.1%)	24 (5.4%)
**on the surface**	27 (100.0%)	420 (95.0%)
**total**	27 (100.0%)	442 (100.0%)
**Buried ASA, (Å²)**	856.4 (36.7%)	811.6 (3.5%)
**Total ASA, (Å²)**	2334.7 (100.0%)	23521.3 (100.0%)
**Solvation energy (kcal/mol)**	-14	-411.7
**SE gain (kcal/mol)**	-4.5	-7.2

Fig. S10. Analysis of the binding interface between OsExo70F2 and AVR-Pii using qtPISA. Interface analysis was performed using qtPISA (19). The key interface parameters in the analysis are represented as an interaction radar and the values are listed in the adjacent table.

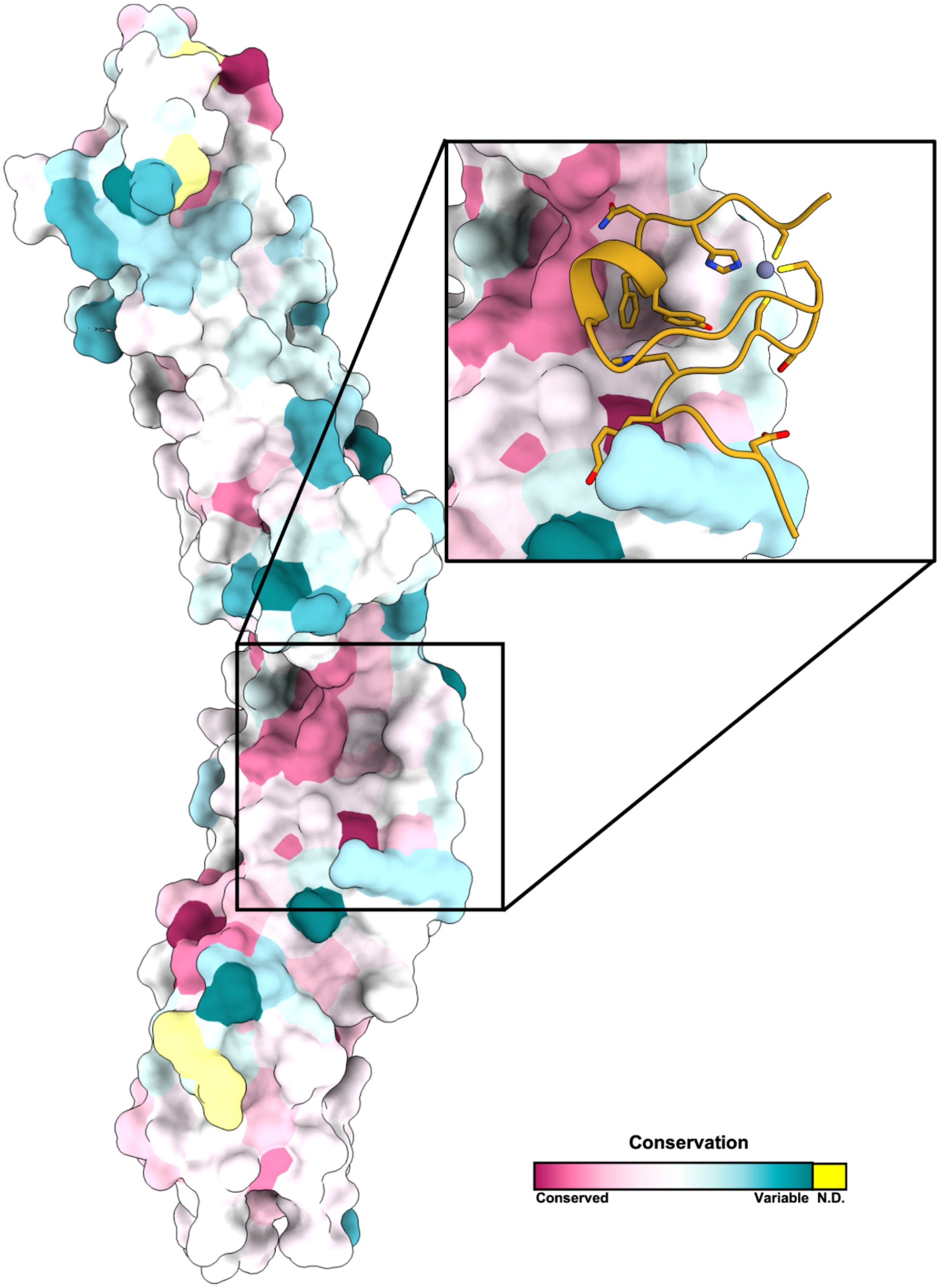

Fig. S11. The amphipathic AVR-Pii binding interface is conserved in rice Exo70s. Conservation profile of rice Exo70 residues with a close-up view at the AVR-Pii interface as calculated by ConSurf (20). Exo70 is represented with solid surface colored according to the conservation of their residues ranging from purple (highly conserved) through white (moderately conserved) to cyan (highly variable). Surface areas highlighted in yellow correspond to residues for which a meaningful conservation level could not be derived from the set of homologues used for the analysis. A close-up view of the effector interface is also shown with AVR-Pii residues 44 to 70 represented in ribbons and colored in yellow with the side chains of important residues displayed as cylinders. Conservation analysis was generated using the rice Exo70 protein sequences reported by Cvrckova et al. (21).

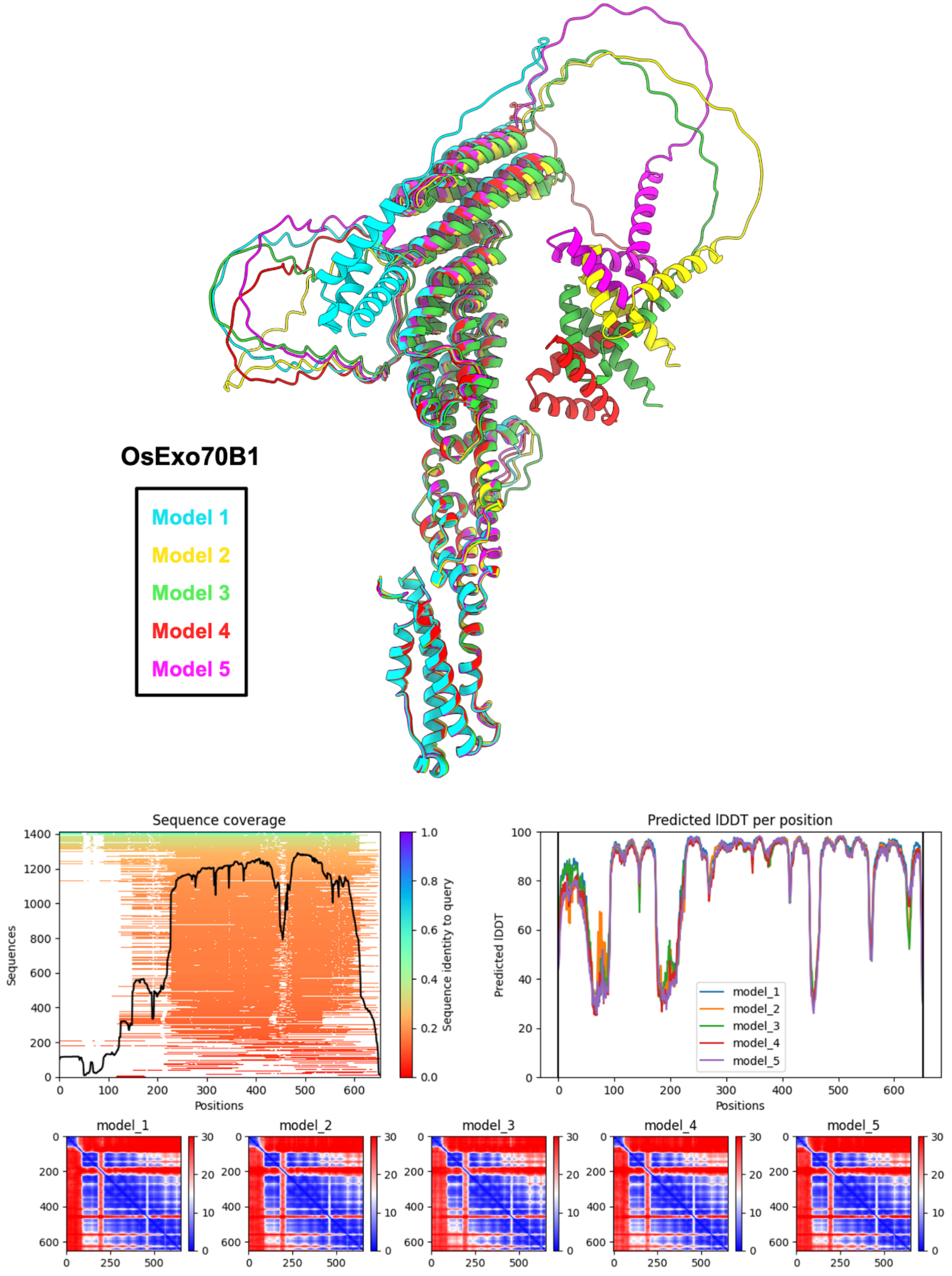

Fig. S12. Homology modelling of OsExo70B1 calculated with AlphaFold2. Structure of OsExo70B1 generated by homology modelling using AlphaFold2 (22) as implemented by ColabFold (23). The five different models generated by default are colored as indicated. Sequence

coverage of the MSA analysis is represented in a graph as number of sequences per position. The calculated confidence score (lDDT) for the positions of each model is also shown.

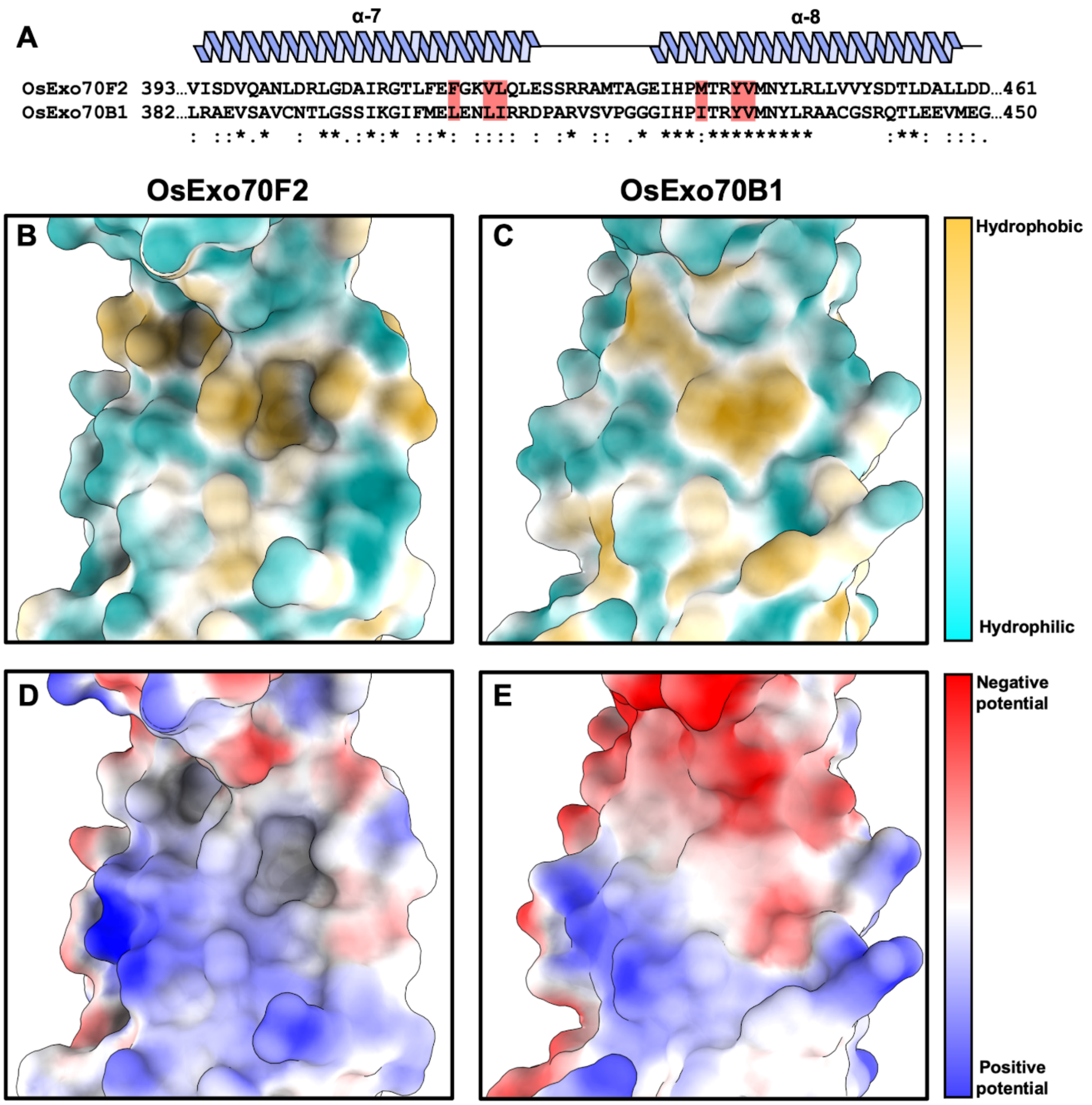

Fig. S13. Comparison of the effector binding interface between OsExo70F2 and OsExo70B1. **(A)** Sequence alignment of residues located at the OsExo70F2 and OsExo70B1 α-helices 7 and 8 generated with Clustal Omega (24). Secondary structure features of Exo70 fold are shown above, and important residues for the formation of the AVR-Pii binding pocket are highlighted in red. Comparison of **(B)** OsExo70F2 and **(C)** OsExo70B1 surface hydrophobicity at the interaction interface with AVR-Pii, residues are colored depending on their hydrophobicity from light blue (low) to yellow (high). Comparison of **(D)** OsExo70F2 and **(E)** OsExo70B1 surface electrostatic potentials at the interaction interface with AVR-Pii, residues are colored depending on their electrostatic potential from dark blue (positive) to red (negative). The OsExo70B1 structure used for comparison was generated using AlphaFold2 (22) **(Figure S12)**.

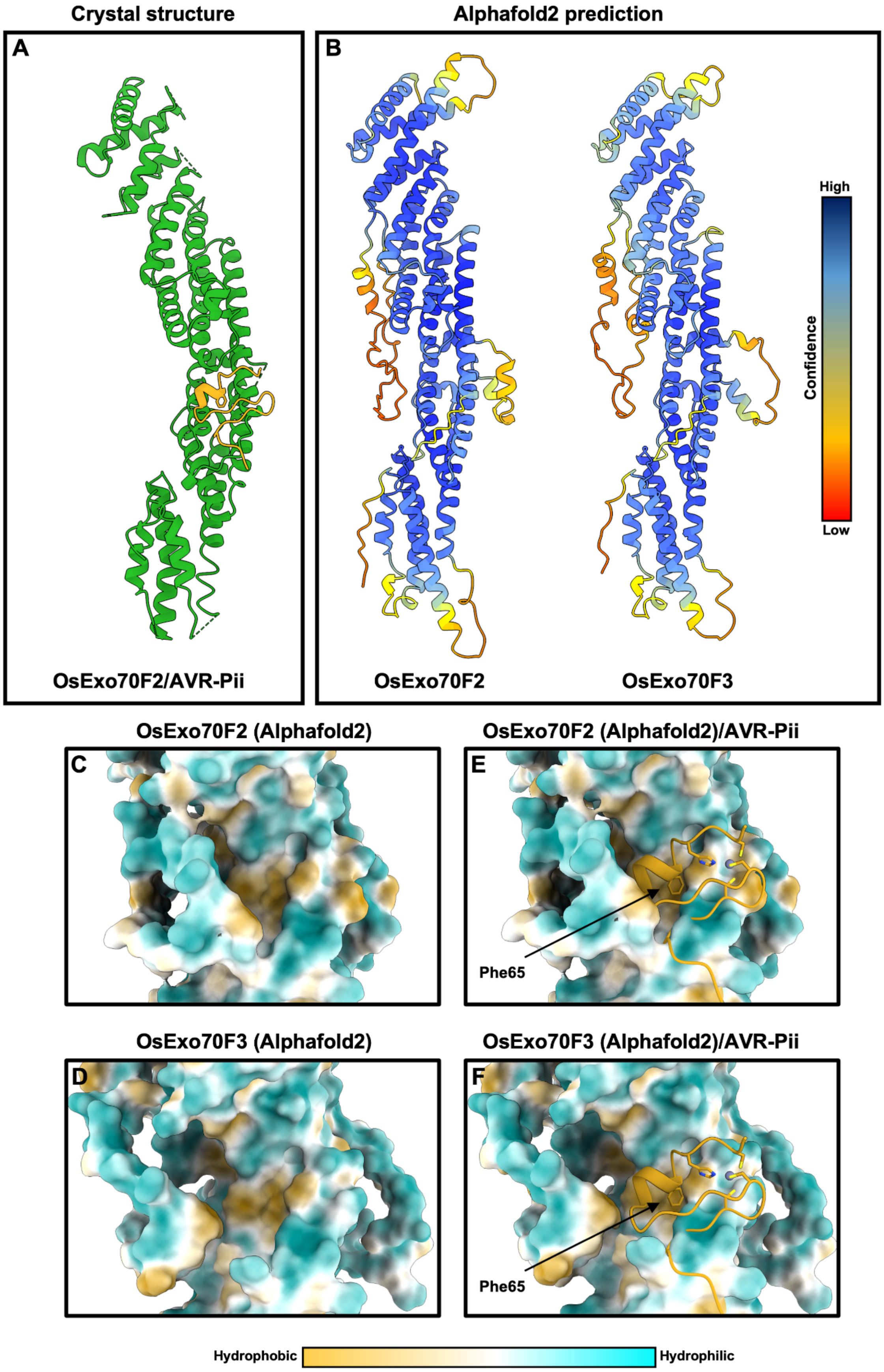

Crystal structure
Alphafold2 prediction
A
B
OsExo70F2/AVR-Pii
OsExo70F2
OsExo70F3
High
Confidence
Low
OsExo70F2 (Alphafold2)
OsExo70F2 (Alphafold2)/AVR-Pii
C
E
Phe65
OsExo70F3 (Alphafold2)
OsExo70F3 (Alphafold2)/AVR-Pii
D
F
Phe65
Hydrophobic
Hydrophilic

Fig. S14. AlphaFold2 predictions of OsExo70F2 and OsExo70F3 displaying the hydrophobic pocket at the AVR-Pii binding interface. (A) The crystal structure of the OsExo70F2/AVR-Pii complex determined in this study. **(B)** AlphaFold2 predictions of OsExo70F2 and OsExo70F3. Models are colored according to confidence (pLDDT) with blue and red regions representing high and low confidence, respectively. **(C-D)** Hydrophobic surface mapping of **(C)** OsExo70F2 and **(D)** OsExo70F3 AlphaFold2 models focusing on the hydrophobic pocket at the AVR-Pii binding interface. **(E-F)** Structural alignment of the crystal structure with the AlphaFold2 models of **(E)** OsExo70F2 and **(F)** OsExo70F3 showing AVR-Pii Phe65 present in the predicted hydrophobic pocket as seen in the crystal structure of the OsExo70F2/AVR-Pii complex.

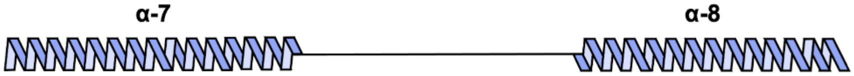


```
OsExo70H3a   266…AGEAARGALANLEVA---IEKEPS---------KATVAGGGVHPLTRYVMNYLVFLA--D…312
OsExo70H3b   274…AGEAARGALANLEVA---IEKEPS---------KATVAGGGVHPLTRYVMNYLVFLA--D…320
OsExo70FX16  313…AGEAARGILVSFEEA---IQKATSKS-------SAAATGGAVHPLARYVMNYLVLLA--D…361
OsExo70H1    313…AGEAARGILVSFEEA---IQKATSKS-------SAAATGGAVHPLARYVMNYLVLLA--D…361
OsExo70H2    289…AGNAARGILMSLEQA---IQKTTS-S-------KAAVTGSAVHPLTRYVMNYLVLLA--D…336
OsExo70I1    403…LARAAAAAFYEFGLR---VETHYVTA-------AAAGESGHVPKIVRYAVNYLKCLASDD…453
OsExo70G1    375…LVDGAVEIFEELLVQ---VELQRHMP---------PPPDGGVPRLVSFVVEYCNRLLSEK…423
OsExo70G2    383…VVDGSVEIFEELLVQ---VELQRNMP---------PPADGGVPRIVSFVAKYCNQLLGDP…431
OsExo70B1    393…LGSSIKGIFMELENL---IRRDPARV---------SVPGGGIHPITRYVMNYLRAAC--G…439
OsExo70B3    402…LGSSIKGIFMELENL---IRRDPARV---------AAQGGGIHPITRYVMNYLRAAC--G…448
OsExo70B2    283…LGLSIKAILMALENL---VRRDPSES---------CPLDGGVHPMTRYVMNYLVTAC--V…329
OsExo70A2    341…LAQTAKKTIGDFMEY---ILNHSVTS---------TTVDGAVHYMTSYVTDYIKFLF--D…387
OsExo70A1    367…LAQTAQKTFSDFEEA---VEKDATKN---------IHIDGTVHPLTSYVINYVKFLF--D…413
OsExo70A3    379…LAQTAQETFADFEEA---VEKDASKT---------IVNDGTVHPLTSYVINYVKFLF--D…425
OsExo70A4    384…LAQTAQETFADFEEA---VEKDTSKT---------IVQDGTVHPLTSYVINYVKFLF--D…430
OsExo70C2    426…LGESAAAIFCDLESS---IRADAGKQ---------PVPGGAVHPLTRYLMNYLKYAC--E…472
OsExo70C1    434…LGEFAAAIFRELESS---IRADAGKQ---------PVPGGAVHPLTRYVMNYLKYAC--E…480
OsExo70E1    223…LGEFAAAIFRELESS---IRADAGKQ---------PVPGGAVHPLTRYVMNYLKYAC--E…269
OsExo70X1    333…LGESVRKTITEFKYA---VQSYTSSN---------AMARGEVHPLTKYVMNYIKALT--A…379
OsExo70D1    349…LADAVRGILSEFENA---VLRDPSKT---------PVPGGTIHPLTRYVMNYSSLIS--D…395
OsExo70D2    343…LADAVRGILSEFENA---VLRDPPKT---------AVPGGTVHPLTRYVMNYSSLIS--D…389
OsExo70F4    325…LGSTIRHTIDQFVNV---IHGESSRR---------PVLGGEIHPMTRYVLNYCGLLA--E…371
OsExo70F1    383…LGDAVRGTVAEFANA---IQGETSRR---------ALPGGEIHPLTRYVMNYVRLLA--D…429
OsExo70F2    404…LGDAIRGTLFEFGKV---LQLESSRR---------AMTAGEIHPMTRYVMNYLRLLV--V…450
OsExo70F3    406…LGDAVKGTLLEFGKV---LQQESSRR---------PMMAGEIHPMTRYVMNYLRLLV--V…452
OsExo70FX13  269…MRNASRGIIDNMKRF---IQHK--------V---QTWDNIAIHPTTCFLINAIKIFN--V…313
OsExo70FX2   288…LCNAIESMAMVAEAVTPVLIGWDS------CENF--KQNAEIHEATRLIVDYVRLFW--G…338
OsExo70FX1   276…LGQAIQRMANDAEAVTPVLSGMHS------WETF--PQSAEIHKATHLIMDYARLFW--G…326
OsExo70FX3   318…LGEAIRKTAKDAEAVTPVLSAMDS------WEKF--TQTEGIHRATRLIVDYASLFW--G…368
OsExo70FX4   280…LSDAIWVMMEKVRAL---FLMDAC------WQVSQEAAASGTHETTELTMNYITLLW--R…329
OsExo70FX5   340…LSDAIWDMMEKVRSS---FLQDGC------WQVSPEASASGVHETTVLMMNYIALLW--R…389
OsExo70FX6   468…SSSTVLQLFMELNNL---IKSQR-----------LVMVDIGVHHITRHITEYMRVLF--E…512
OsExo70FX7   450…LSAVFLVLLVELNGL---LRSQH-----------LVISNTGVHHVTQHIMGLMRLLV--E…494
OsExo70FX11  439…LGVAVRGILSKTKAL---IHSYGGSPGQNVVVVVVVPDGGGIHVVTSYLARYVELLA--Q…494
OsExo70FX10  458…LEDSAREILGKLKVQ---IQSHDS---------PII-PGGSVHLVTTYLMRYITLLA--H…503
OsExo70FX12  415…LKDGTKETLDQLRVQ---IQSYSS---------EDMPEGGGIHLVTTYLIRYIMSLT--Q…461
OsExo70FX9   240…LVDAARRCVRGLPAS---IRSHY---------PWRMPQGGEVHPCVGFWMGYFRCML--R…286
OsExo70FX14  338…LARALRAMIGGLIAR---IRKADCPQT-----TGSAARGVGVHPLTRYAMTCVELLS--P…388
OsExo70FX15  319…LAMSIRSMVASLIAK---VRDGVS--------NTKNIVGVGVHPLTKYAVLCIVRLA--P…366
```

Fig. S15. Alignment of OsExo70 proteins showing subtle differences in residues that form the binding interface with AVR-Pii. Protein sequence alignment of rice Exo70 proteins reported by Cvrckova et al. (21) generated with Clustal Omega (24), focusing on OsExo70F2 residues that interact with AVR-Pii. OsExo70 proteins studied here are highlighted in blue. Equivalent positions of OsExo70 residues contributing to the formation of the hydrophobic pocket in OsExo70F2 are highlighted in red.

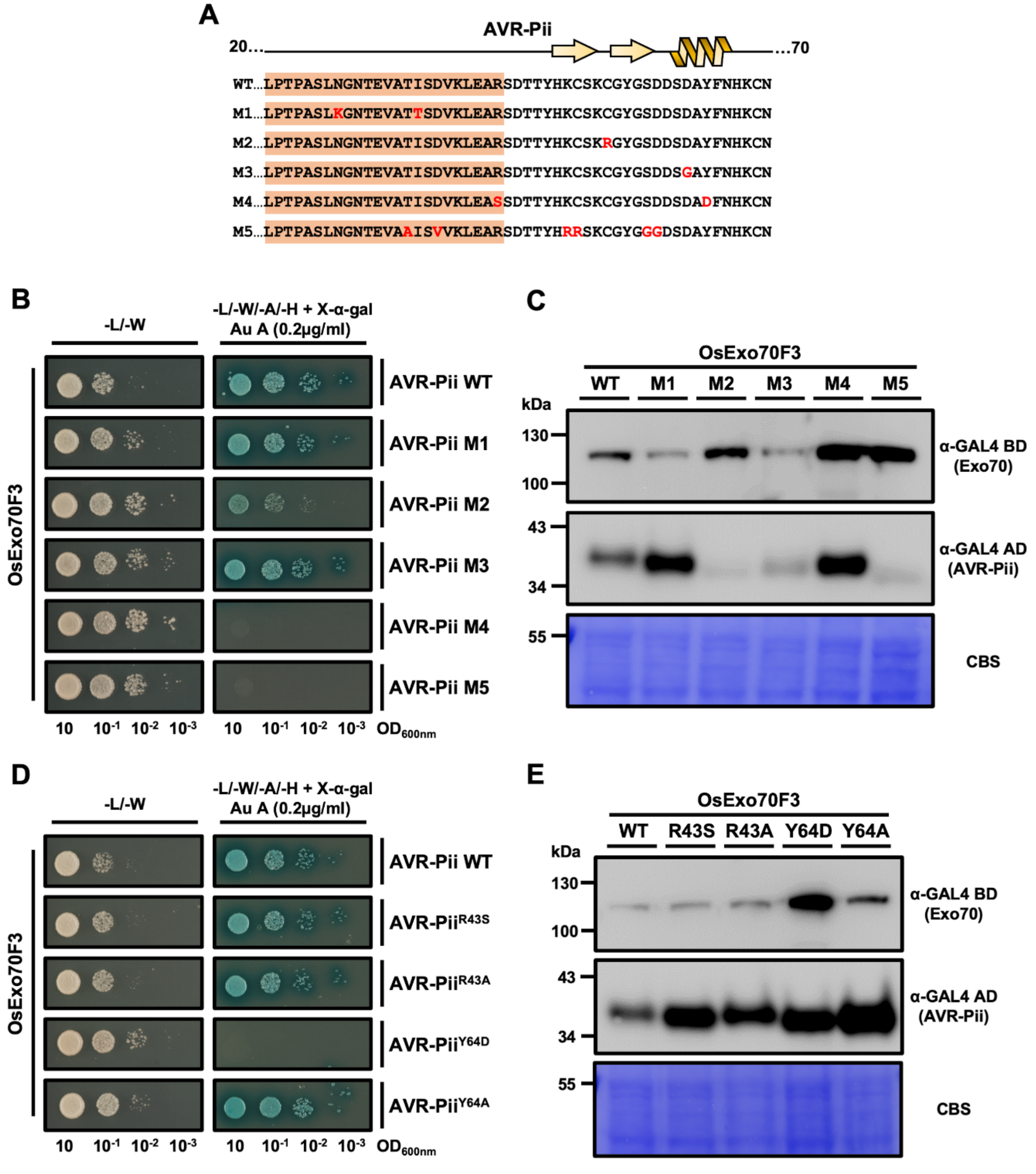

Fig. S16. Random mutagenesis identified AVR-Pii residues that alter binding to OsExo70F3. **(A)** Amino acid sequence of the AVR-Pii mutants obtained by random mutagenesis. Secondary structure features of the AVR-Pii fold are shown above, and the residues not observed in the crystal structure are highlighted in orange. **(B)** Yeast-Two-Hybrid assay of AVR-Pii mutants obtained by random mutagenesis with OsExo70F3. The control plate for yeast growth is on the left, with quadruple dropout media supplemented with X-α-gal and Aureobasidine A (Au A) on the right. Growth and development of blue coloration in the selection plate are both indicative of protein:protein interactions. Wild-type AVR-Pii is included as positive control. OsExo70F3 was fused to the GAL4 DNA binding domain, and AVR-Pii mutants to the GAL4 activator domain. Each experiment was repeated a minimum of three times, with similar results. **(C)** Accumulation of AVR-Pii mutants in Yeast-Two-Hybrid assays analyzed by Western blot. Yeast lysate was probed for the

expression of OsExo70F3 and AVR-Pii mutants obtained by random mutagenesis using anti-GAL4 binding domain (BD) and anti-GAL4 DNA activation domain (AD) antibodies, respectively. Total protein extracts were colored with Coomassie Blue Stain (CBS). **(D)** Deconvolution of residues involved in AVR-Pii binding by Yeast-Two-Hybrid assay with rice Exo70F3. The control plate for yeast growth is on the left, with quadruple dropout media supplemented with X-α-gal and Aureobasidine A (Au A) on the right. Growth and development of blue coloration in the selection plate are both indicative of protein:protein interactions. Wild-type AVR-Pii is included as positive control. OsExo70F3 was fused to the GAL4 DNA binding domain, and AVR-Pii point mutants to the GAL4 activator domain. Each experiment was repeated a minimum of three times, with similar results. **(E)** Accumulation of AVR-Pii point mutants in yeast-two-hybrid assays analyzed by Western blot. Yeast lysate was probed for the expression of OsExo70F3 and AVR-Pii point mutants using anti-GAL4 binding domain (BD) and anti-GAL4 DNA activation domain (AD) antibodies, respectively. Total protein extracts were colored with Coomassie Blue Stain (CBS).

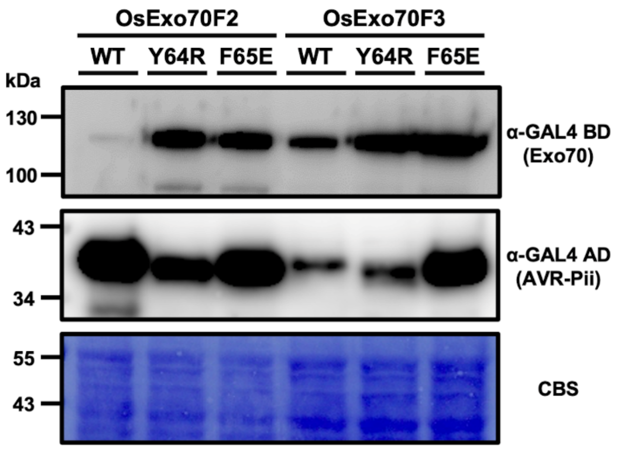

Fig. S17. Accumulation of proteins in Yeast-Two-Hybrid assays analyzed by Western blot. Yeast lysate was probed for the expression of rice Exo70 proteins and AVR-Pii wild-type, AVR-Pii Tyr64Arg and AVR-Pii Phe65Glu using anti-GAL4 binding domain (BD) and anti-GAL4 DNA activation domain (AD) antibodies, respectively. Total protein extracts were colored with Coomassie Blue Stain (CBS).

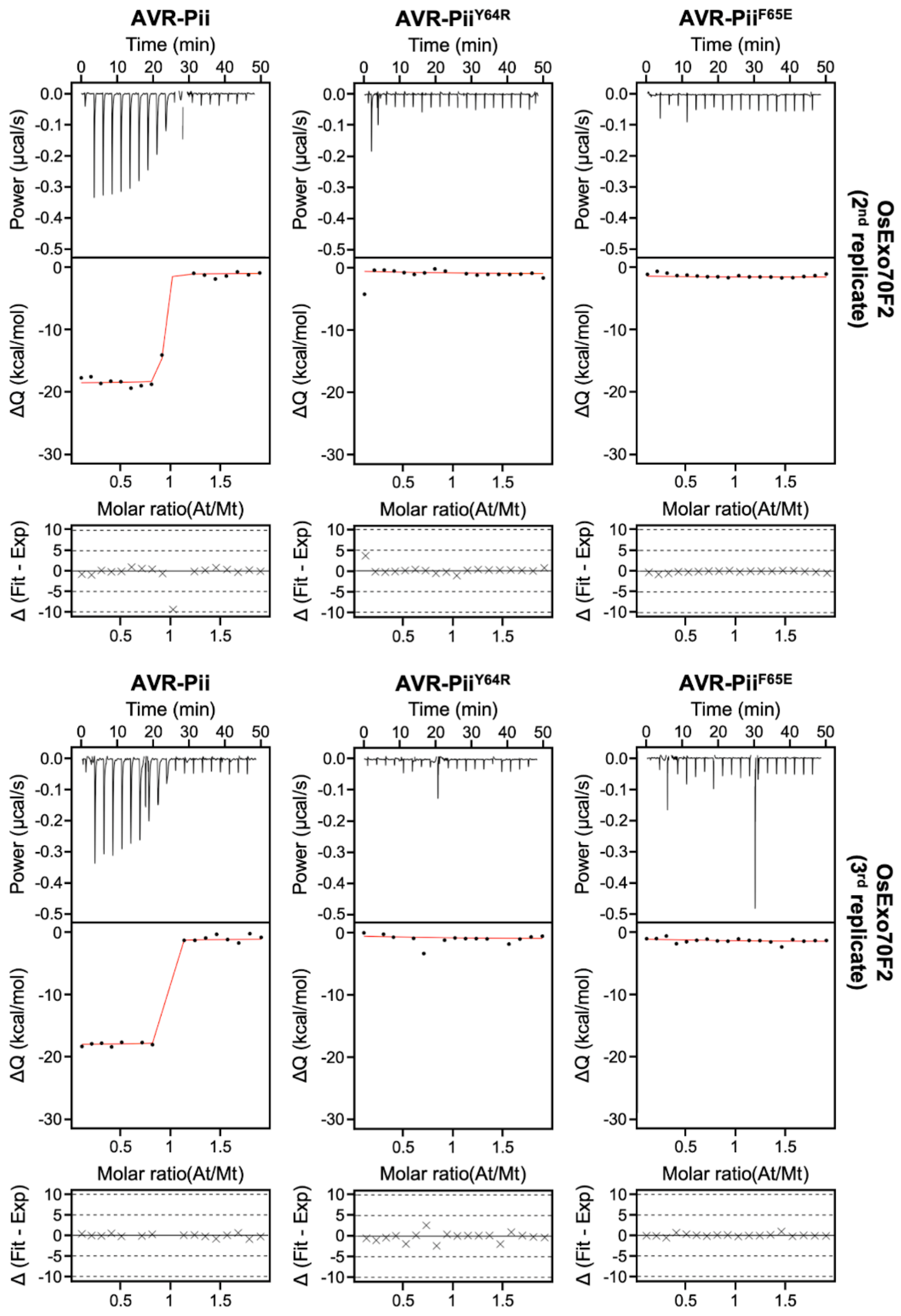

Fig. S18. Experimental replicates for the binding of AVR-Pii mutants to OsExo70F2 measured by ITC. Upper panels show heat differences upon injection of AVR-Pii mutants into the cell containing OsExo70F2. Middle panels show integrated heats of injection (dots) and the best fit (solid line) using to a single site binding model calculated using AFFINImeter ITC analysis software (16). Bottom panels represent the difference between the fit to a single site binding model and the experimental data; the closer to zero indicates stronger agreement between the data and the fit. The thermodynamic parameters obtained in each experiment are presented in **Table S3**.

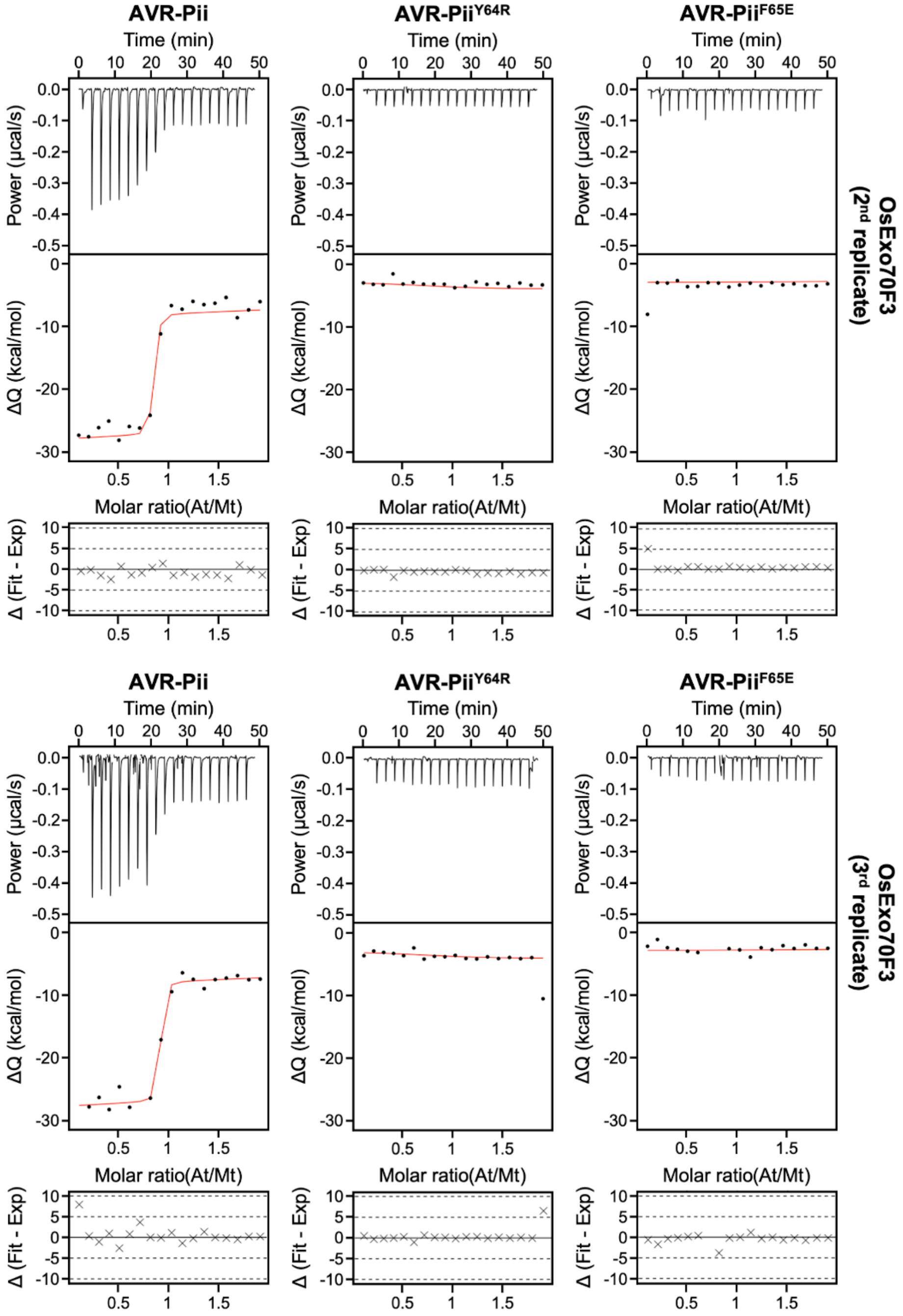

Fig. S19. Experimental replicates for the binding of AVR-Pii mutants to OsExo70F3 measured by ITC. Upper panels show heat differences upon injection of AVR-Pii mutants into the cell containing OsExo70F3. Middle panels show integrated heats of injection (dots) and the best fit (solid line) using to a single site binding model calculated using AFFINImeter ITC analysis software (16). Bottom panels represent the difference between the fit to a single site binding model and the experimental data; the closer to zero indicates stronger agreement between the data and the fit. The thermodynamic parameters obtained in each experiment are presented in **Table S3**.

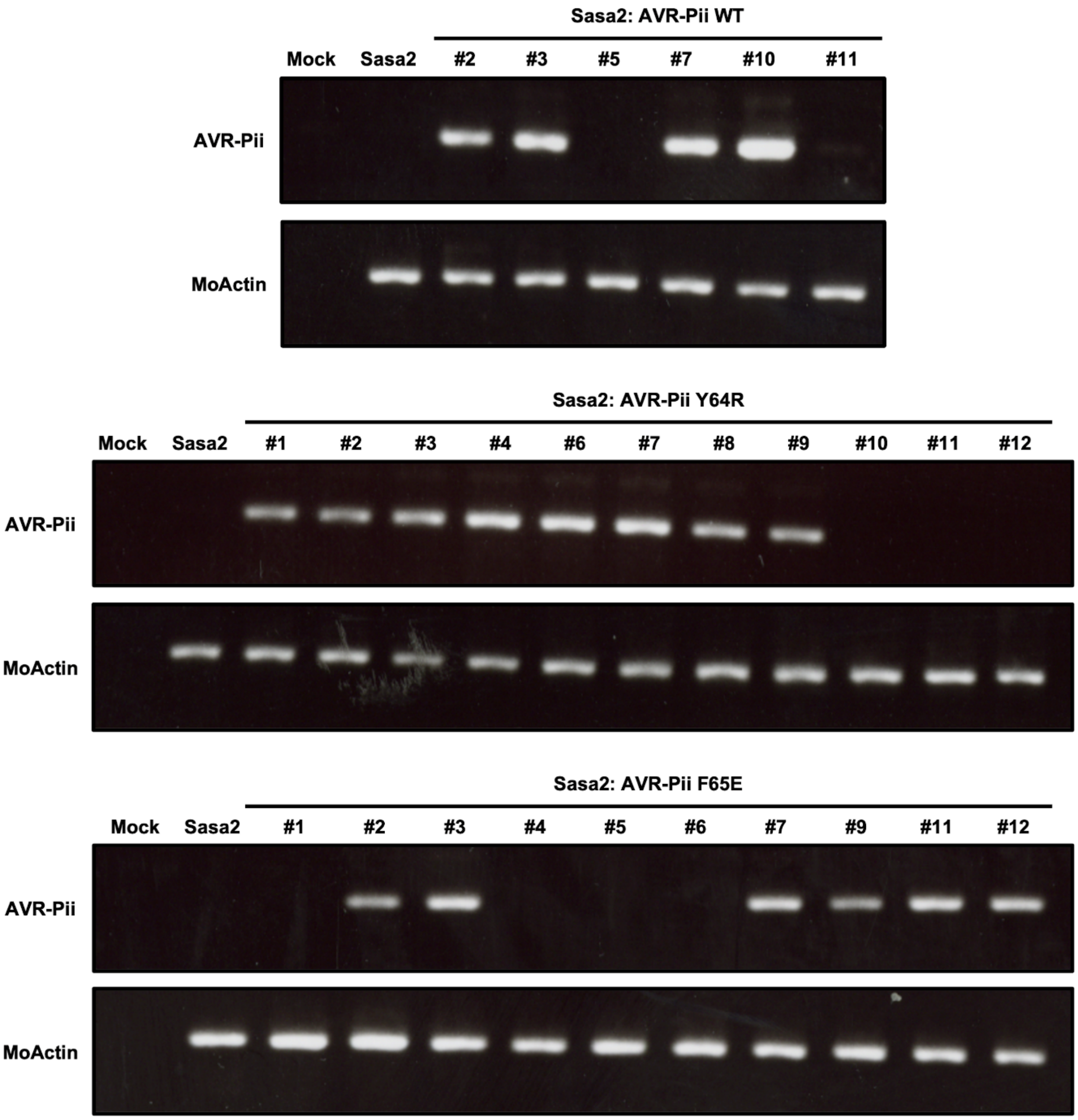

Fig. S20. RT-PCR analysis of effector expression in Sasa2 isolates transformed with AVR-Pii and mutants. The presence of a band depicts that the gene is expressed in rice leaves during infection by the fungal isolates. The top panel corresponds to the expression of AVR-Pii WT or mutants. The expression of MoActin was tested as a positive control and is shown in the bottom panel.

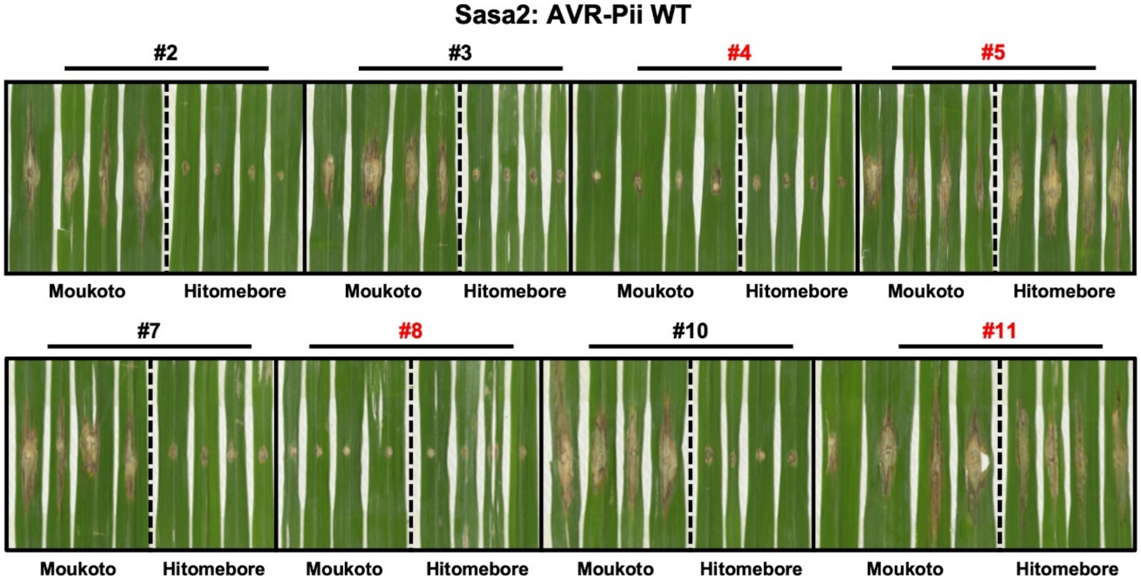

Fig. S21. Replicates of the disease resistance assays of Sasa2 harbouring AVR-Pii WT. Rice leaf blade spot inoculation of transgenic *M. oryzae* Sasa2 into rice cultivars Moukoto (Pii-) and Hitomebore (Pii+). Eight independent Sasa2 transformants harboring wild-type AVR-Pii were spotted in both cultivars. Line numbers colored in red indicate the transformants removed from further quantification because they did not express AVR-Pii effectors as tested by RT-PCR **(Figure S20)** or were not infective.

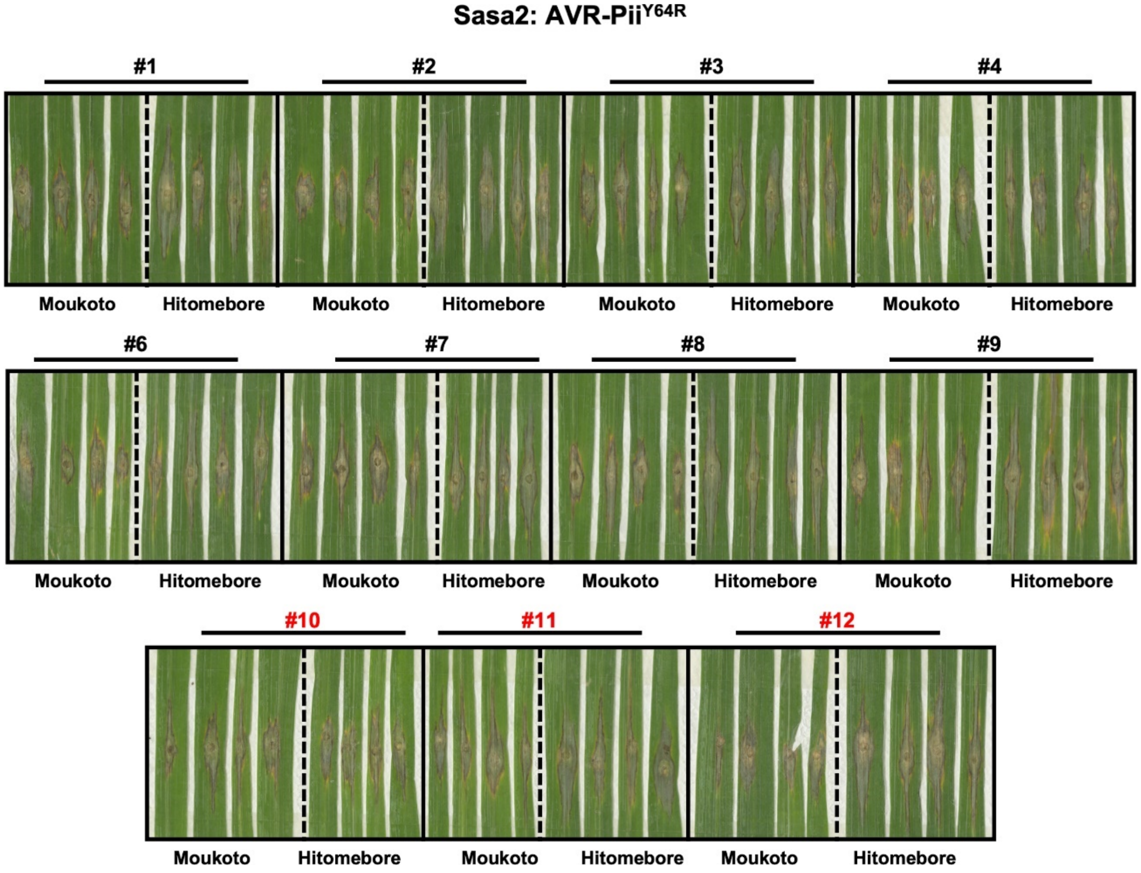

Fig. S22. Replicates of the disease resistance assays of Sasa2 harbouring AVR-Pii Tyr64Arg. Rice leaf blade spot inoculation of transgenic *M. oryzae* Sasa2 into rice cultivars Moukoto (Pii-) and Hitomebore (Pii+). Eleven independent Sasa2 transformants harboring mutant AVR-Pii Tyr64Arg were spotted in both cultivars. Line numbers colored in red indicate the transformants removed from further quantification because they did not express AVR-Pii effectors as tested by RT-PCR **(Figure S20)** or were not infective.

Sasa2: AVR-Pii^{F65E}

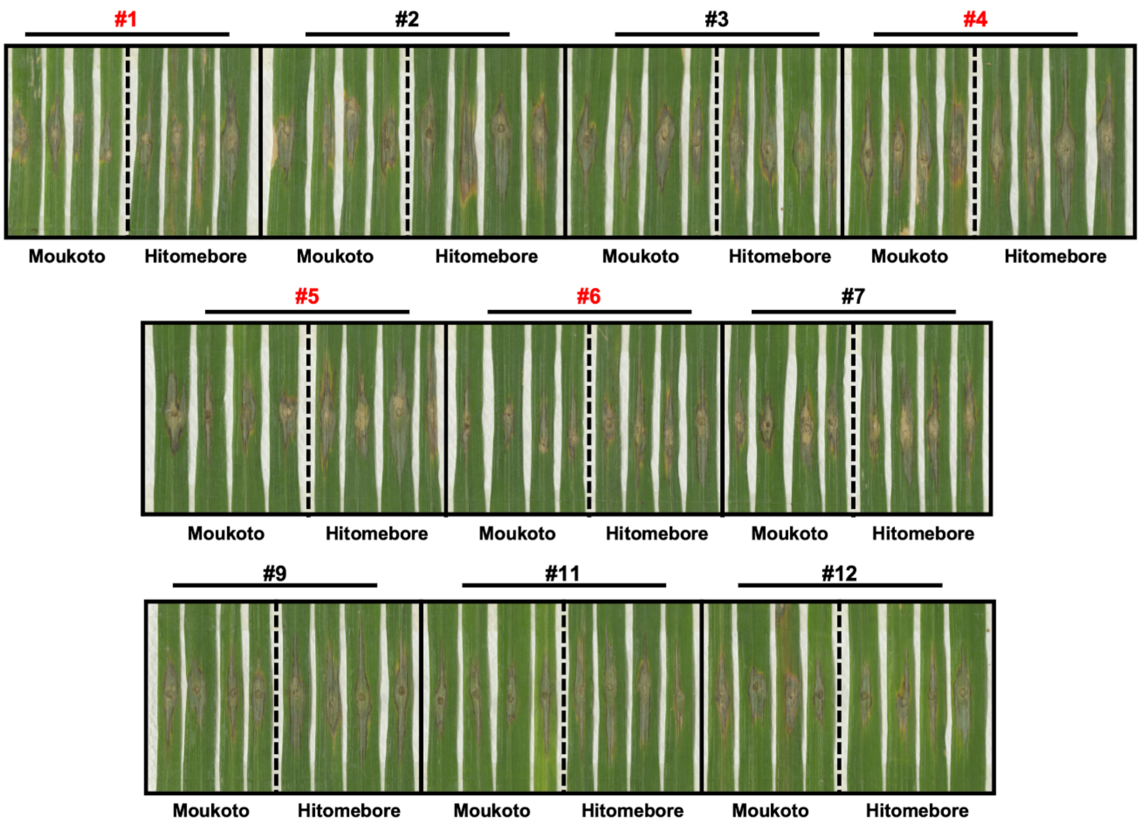

Fig. S23. Replicates of the disease resistance assays of Sasa2 harbouring AVR-Pii Phe65Glu. Rice leaf blade spot inoculation of transgenic *M. oryzae* Sasa2 into rice cultivars Moukoto (Pii-) and Hitomebore (Pii+). Ten independent Sasa2 transformants harboring mutant AVR-Pii Phe65Glu were spotted in both cultivars. Line numbers colored in red indicate the transformants removed from further quantification because they did not express AVR-Pii effectors as tested by PCR **(Figure S20)** or were not infective.

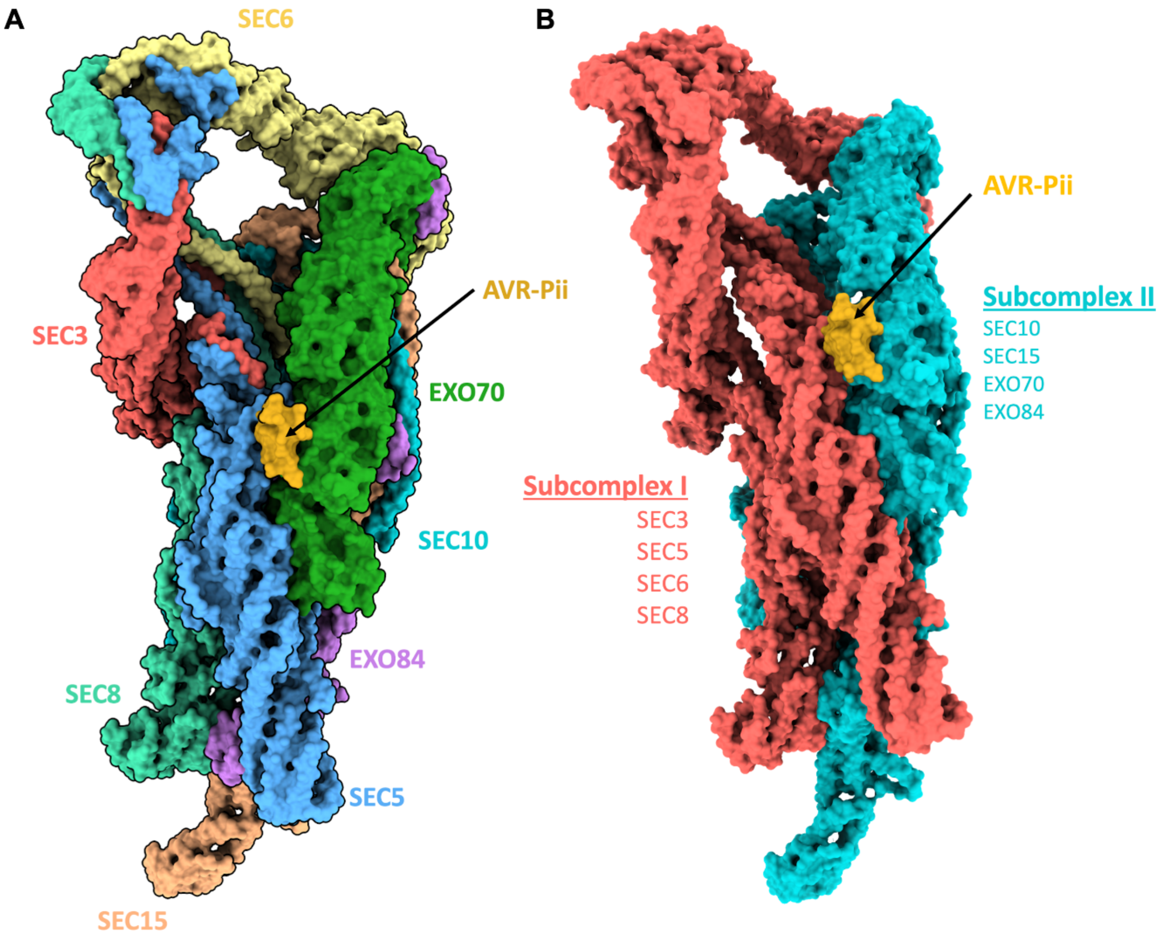

Fig. S24. Representation of AVR-Pii interaction in the context of the exocyst complex. Structural alignment of AVR-Pii/OsExo70F2 structure in the Cryo-EM model of the yeast exocyst complex (PDB ID: 5YFP). The effector is colored in gold and pointed by an arrow while the exocyst complex subunits are **(A)** individually colored or **(B)** colored according to the organization in subcomplexes.

Table S1. Experimental details and thermodynamic parameters obtained from ITC experiments presented in **Figure 1**.

Cell	Conc. [μM]	Syringe	Conc. [μM]	T (℃)	ΔH (kcal mol^{-1}])	K_d (nM)
OsExo70B1	10	AVR-Pii	100	25	n.b.	n.b
OsExo70F2	10	AVR-Pii	100	25	-24.04 ± 0.024	< 1
OsExo70F3	10	AVR-Pii	100	25	-17.67 ± 0.007	4.19

Table S2. Data collection and refinement statistics. (*) The highest resolution shell is shown in parenthesis. (**) As calculated by MolProbity.

OsExo70F2/AVR-Pii	
**Data collection statistics**	
Wavelength (Å)	0.97625
Space group	*C*2
Cell dimensions	104.3 76.6 67.9
a, b, c, α, β, γ (Å, º)	90 107.8 90
Resolution (Å)*	66.81-2.69 (2.82-2.69)
R_{merge} (%)	14.4 (170.9)
$I/\sigma I$	6.7 (1.3)
Completeness (%)	
Overall	99.9 (99.7)
Anomalous	99.7 (99.4)
Unique reflections	19153 (2534)
Redundancy	6.7 (6.5)
CC(1/2) (%)	99.7 (54.1)
**Refinement and model statistics**	
Resolution (Å)	66.81-2.69 (2.82-2.69)
R_{work}/R_{free} (%)	25.2/27.9
No. atoms	
Protein	3738
Ligand	1
Average B-factors	
Protein	87.0
Ligand	62.0
R.m.s deviations	
Bond lengths (Å)	0.0133
Bond angles (º)	1.64
Ramachandran plot (%)**	
Favoured	96.2
Allowed	3.8
Outliers	0
MolProbity Score	1.78

Table S3. Experimental details and thermodynamic parameters obtained from ITC experiments presented in **Figure 3**.

Cell	Conc. [μM]	Syringe	Conc. [μM]	T (℃)	ΔH (kcal mol^{-1}])	K_d (nM)
OsExo70F2	10	AVR-Pii	100	25	-15.67 ± 0.027	<1
OsExo70F2	10	AVR-Pii^{Y64R}	100	25	n.b.	n.b.
OsExo70F2	10	AVR-Pii^{F65E}	100	25	n.b.	n.b.
OsExo70F3	10	AVR-Pii	100	25	-19.01 ± 0.084	2.787
OsExo70F3	10	AVR-Pii^{Y64R}	100	25	n.b.	n.b.
OsExo70F3	10	AVR-Pii^{F65E}	100	25	n.b.	n.b.

SI References

1. Berrow NS*, et al.* (2007) A versatile ligation-independent cloning method suitable for high-throughput expression screening applications. *Nucleic Acids Res* 35(6):e45.
2. Fujisaki K*, et al.* (2015) Rice Exo70 interacts with a fungal effector, AVR-Pii, and is required for AVR-Pii-triggered immunity. *Plant J* 83(5):875-887.
3. Yoshida K*, et al.* (2009) Association genetics reveals three novel avirulence genes from the rice blast fungal pathogen Magnaporthe oryzae. *The Plant cell* 21(5):1573-1591.
4. Sweigard JA, F. Chumley, A. Carroll, L. Farrall, and B. Valent (1997) A series of vectors for fungal transformation. *Fungal Genetics Reports* 44.
5. Studier FW (2005) Protein production by auto-induction in high density shaking cultures. *Protein Expr Purif* 41(1):207-234.
6. Lobstein J*, et al.* (2012) SHuffle, a novel Escherichia coli protein expression strain capable of correctly folding disulfide bonded proteins in its cytoplasm. *Microb Cell Fact* 11:56.
7. Winter G (2010) xia2: an expert system for macromolecular crystallography data reduction. *Journal of Applied Crystallography* 43(1):186-190.
8. Winn MD*, et al.* (2011) Overview of the CCP4 suite and current developments. *Acta Crystallogr D Biol Crystallogr* 67(Pt 4):235-242.
9. McCoy AJ*, et al.* (2007) Phaser crystallographic software. *J Appl Crystallogr* 40(Pt 4):658-674.
10. Cowtan K (2006) The Buccaneer software for automated model building. 1. Tracing protein chains. *Acta Crystallographica Section D* 62(9):1002-1011.
11. Emsley P, Lohkamp B, Scott WG, & Cowtan K (2010) Features and development of Coot. *Acta Crystallogr D Biol Crystallogr* 66(Pt 4):486-501.
12. Murshudov GN*, et al.* (2011) REFMAC5 for the refinement of macromolecular crystal structures. *Acta Crystallographica Section D* 67(4):355-367.
13. Croll T (2018) ISOLDE: a physically realistic environment for model building into low-resolution electron-density maps. *Acta Crystallographica Section D* 74(6):519-530.
14. Pettersen EF*, et al.* (2021) UCSF ChimeraX: Structure visualization for researchers, educators, and developers. *Protein science : a publication of the Protein Society* 30(1):70-82.
15. Chen VB*, et al.* (2010) MolProbity: all-atom structure validation for macromolecular crystallography. *Acta Crystallogr D Biol Crystallogr* 66(Pt 1):12-21.
16. Piñeiro Á*, et al.* (2019) AFFINImeter: A software to analyze molecular recognition processes from experimental data. *Analytical Biochemistry* 577:117-134.
17. Zhang C*, et al.* (2016) Endosidin2 targets conserved exocyst complex subunit EXO70 to inhibit exocytosis. *Proceedings of the National Academy of Sciences* 113(1):E41-E50.
18. Moore BA, Robinson HH, & Xu Z (2007) The crystal structure of mouse Exo70 reveals unique features of the mammalian exocyst. *Journal of molecular biology* 371(2):410-421.

19. Krissinel E (2015) Stock-based detection of protein oligomeric states in jsPISA. *Nucleic Acids Res* 43(W1):W314-319.
20. Ashkenazy H*, et al.* (2016) ConSurf 2016: an improved methodology to estimate and visualize evolutionary conservation in macromolecules. *Nucleic Acids Res* 44(W1):W344-350.
21. Cvrckova F*, et al.* (2012) Evolution of the Land Plant Exocyst Complexes. *Frontiers in Plant Science* 3(159).
22. Jumper J*, et al.* (2021) Highly accurate protein structure prediction with AlphaFold. *Nature* 596(7873):583-589.
23. Mirdita M*, et al.* (2022) ColabFold: making protein folding accessible to all. *Nat Methods* 19(6):679-682.
24. Sievers F & Higgins DG (2014) Clustal Omega, accurate alignment of very large numbers of sequences. *Methods in molecular biology (Clifton, N.J.)* 1079:105-116.
